# Synthesis of 2-substituted tetraphenylenes via transition-metal-catalyzed derivatization of tetraphenylene

**DOI:** 10.3762/bjoc.12.122

**Published:** 2016-06-22

**Authors:** Shulei Pan, Hang Jiang, Yanghui Zhang, Yu Zhang, Dushen Chen

**Affiliations:** 1 Department of Chemistry, and Shanghai Key Lab of Chemical Assessment and Sustainability, Tongji University, 1239 Siping Road, Shanghai, 200092, P. R. China

**Keywords:** acetoxylation, carbonylation, halogenation, tetraphenylene, transition metal

## Abstract

A new strategy for the synthesis of 2-substituted tetraphenylenes through a transition-metal-catalyzed derivatization has been developed. Three types of functionalities, including OAc, X (Cl, Br, I) and carbonyl, were introduced onto tetraphenylene, which allows the easy access to a variety of monosubstituted tetraphenylenes. These reactions could accelerate research on the properties and application of tetraphenylene derivatives.

## Introduction

**Figure 1:**
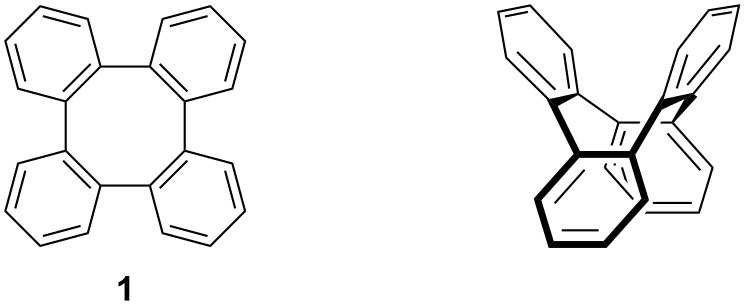
Tetraphenylene and its saddle-shaped structure.

Tetraphenylene (**1**) is one of the simplest motifs in the eight-membered ring aromatic compounds (Figure 1) [1–2]. Based on its nonplanar distinct saddle-shaped structure [3–4], tetraphenylene and its derivatives have found broad applications in materials science [5–11], supramolecular chemistry [12–18], and asymmetric catalysis [19–21].

Since Rapson and co-workers reported the first synthesis of tetraphenylene in 1943 [22], in which 2,2’-dibromobiphenyl was converted to its corresponding Grignard reagent and subsequent addition of copper(II) chloride provided **1** in 16% yield, a variety of methods for constructing the tetraphenylene skeleton have been developed [23–42]. While most of these traditional approaches suffer from harsh conditions or complicated procedures, a novel strategy via transition-metal-catalyzed C–H activation has attracted great attention and emerged as a powerful methodology for the synthesis of tetraphenylenes [43–44]. However, the methods of this strategy have a relatively limited substrate scope and are primarily applicable to the synthesis of symmetrically substituted tetraphenylenes. Among the various reactions that have been developed for the construction of the tetraphenylene skeleton, the methods for the synthesis of tetraphenylene derivatives via the direct derivatization of tetraphenylene are rare. More importantly, the direct derivatization of tetraphenylene would provide an efficient method for the synthesis of tetraphenylene derivatives, in particular for unsymmetrically substituted ones. Although direct bromination [22], nitration [22], and acetylation [45] of tetraphenylene via electrophilic aromatic substitution have been reported, it is still desirable to develop new methods for the derivatization of tetraphenylenes. Herein we report several synthetic protocols for the transition-metal-catalyzed derivatization of tetraphenylene, which provide a new method for the synthesis of 2-substituted tetraphenylenes.

## Results and Discussion

The acetoxy group is an important functional group because it can be transformed into a variety of other functionalities [46–47], thus making the acetoxylation a highly interesting reaction. The Sanford and Wang group, respectively, developed a highly efficient palladium and gold-catalyzed direct acetoxylation of arenes with iodobenzene diacetate [48–49]. Based on these excellent works, we surveyed the reaction conditions for the acetoxylation of tetraphenylene (**1**). For this, **1** was allowed to react with PhI(OAc)_2_ (**2a**) in the presence of Pd(OAc)_2_/pyridine as catalysis system in a mixture of AcOH and Ac_2_O at 100 °C. Gratefully, the desired acetylated product **3a** was formed in 52% yield (Table 1, entry 1). Prolonging the reaction time or carrying out the reaction at 120 °C led to lower yields (Table 1, entries 2 and 3). However, the yields increased when increasing amounts of PhI(OAc)_2_ were used (58% for 3.0 equiv and 70% for 4.0 equiv, respectively). Further increase of PhI(OAc)_2_ beyond 4.0 equiv failed to further improve the yield. On the other hand, the yield increased to 75% by using higher concentrations of the reactants.

**Table 1:** The Pd(OA)_2_-catalyzed acetoxylation of tetraphenylene (**1**).

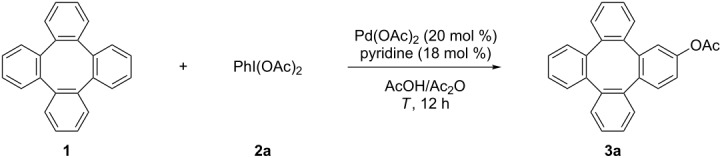				

Entry	PhI(OAc)_2_ (equiv)	Temperature, *T* (°C)	AcOH/Ac_2_O (mL)	Yield^a^ (%)

1	2.0	100	0.90:0.10	52
2	2.0	100	0.90:0.10	42^b^
3	2.0	120	0.90:0.10	34
4	3.0	100	0.90:0.10	58
5	4.0	100	0.90:0.10	70
**6**	**3.0**	**100**	**0.45:0.05**	**75 (72)**^c^
7	4.0	100	0.45:0.05	74

^a^The yields were determined by ^1^H NMR analysis of the crude products using CH_2_Br_2_ as the internal standard. ^b^Reaction time 24 h. ^c^Isolated yields based on tetraphenylene (**1**).

Halogen-substituted compounds are another important substance class in organic synthesis, since these substituents allow access to a variety of functionalities [50–55]. In this context especially the direct halogenation of tetraphenylene attracted our attention. The Wang group reported an efficient and mild protocol for a gold-catalyzed direct C–H halogenation of arenes with *N*-halosuccinimides [56–57]. Therefore, we initially investigated the chlorination of tetraphenylene by subjecting it to Wang’s conditions, and the reaction gave **3b** in 28% (Table 2, entry 1). The yield decreased when the reaction was carried out at a lower or higher temperature (Table 2, entries 2 and 3). Gratefully, the yield was dramatically enhanced to 72% when 0.4 equiv of BF_3_·Et_2_O was added (Table 2, entry 4), and further to 90% when the reaction was run for 24 hours (Table 2, entry 5). Finally, the optimal 94% yield was achieved using 2.0 equiv NCS (Table 2, entry 6).

**Table 2:** The AuCl_3_-catalyzed chlorination of tetraphenylene (**1**).

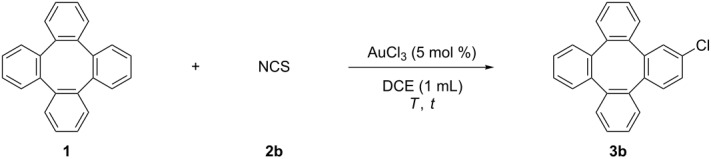					

Entry	NCS (equiv)	Additive (equiv)	*T* (°C)	*t* (h)	Yield^a^ (%)

1	1.0	–	80	12	28
2	1.0	–	100	12	26
3	1.0	–	60	24	24
4	1.0	BF_3_·Et_2_O (0.4)	80	12	72
5	1.0	BF_3_·Et_2_O (0.4)	80	24	90
**6**	**2.0**	**BF_3_**·**Et_2_O (0.4)**	**80**	**24**	**94 (91)**^b^

^a^The yields were determined by ^1^H NMR analysis of the crude products using CH_2_Br_2_ as the internal standard. ^b^Isolated yields based on tetraphenylene (**1**).

Subsequently, the bromination of tetraphenylene (**1**) was examined. The reaction yielded the desired brominated product **3c** under Wang’s conditions in 64% yield (Table 3, entry 1). Increasing or lowering the temperature again failed to improve the yield (Table 3, entries 2 and 3). The yield increased to 86% when 1.5 equiv NBS was used (Table 3, entry 4), and was further optimized to 98% when the reaction time was prolonged to 24 hours (Table 3, entry 5).

**Table 3:** The AuCl_3_-catalyzed bromination of tetraphenylene (**1**).

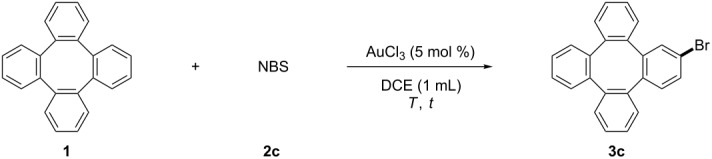				

Entry	NBS (equiv)	*T* (°C)	*t* (h)	Yield^a^ (%)

1	1.0	80	12	64
2	1.0	50	12	54
3	1.0	100	12	64
4	1.5	80	12	86
**5**	**1.5**	**80**	**24**	**98 (95)**^b^

^a^The yields were determined by ^1^H NMR analysis of the crude products using CH_2_Br_2_ as the internal standard. ^b^Isolated yields based on tetraphenylene (**1**).

Next, we surveyed the reaction conditions for the iodination of tetraphenylene with NIS (**2d**). Under the reaction conditions developed by the Wang group, the desired iodinated tetraphenylene **3d** was obtained in 18% yield as shown in Table 4 (entry 1). When the reaction was performed at 60 °C, the yield of **3d** increased to 32% (Table 4, entry 2). However, further enhancing the temperature failed to give a higher yield (Table 4, entry 3). The addition of 2.0 equiv **2d** improved the yield remarkably (Table 4, entry 4). The yields decreased when the reaction time was shortened or prolonged (Table 4, entries 5 and 6).

**Table 4:** The AuCl_3_-catalyzed iodination of tetraphenylene (**1**).

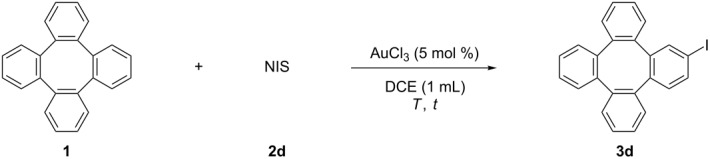				

Entry	NIS (equiv)	*T* (°C)	*t* (h)	Yield^a^ (%)

1	1.0	rt	12	18
2	1.0	60	12	32
3	1.0	80	12	30
**4**	**2.0**	**60**	**12**	**80 (78)**^b^
5	2.0	60	6	72
6	2.0	60	16	52

^a^The yields were determined by ^1^H NMR analysis of the crude products using CH_2_Br_2_ as the internal standard. ^b^Isolated yields based on tetraphenylene (**1**).

Having successfully developed protocols for introducing OAc and X (Cl, Br, I) onto tetraphenylene (**1**), we next turned to investigate the carbonylation of tetraphenylene (**1**). The carbonyl group is a common structural element present in both natural products and functional materials and can be transformed into other functionalities [58–59]. The Larock group reported a novel Pd-catalyzed addition of nitriles to an arene C–H bond for the synthesis of aryl ketones [60–61]. Following the Larock’s conditions, we investigated the carbonylation of tetraphenylene (**1**) and the carbonylated product **5a** was obtained in 20% yield (Table 5, entry 1). While the yield was improved to 42% in the presence of 2.0 equiv DMSO (Table 5, entry 2), it decreased when 4.0 equiv DMSO were used (Table 5, entry 3). Since the solubility of **1** in trifluoroacetic acid is low, we envisaged that the addition of co-solvents would promote the reaction. Therefore, we screened the effect of different co-solvents on the reaction and dichloromethane was found to be the best choice (Table 5, entries 4 and 5). The yield was remarkably improved by increasing the amount of Pd(OAc)_2_ (Table 5, entry 6). Raising or lowering the temperature led to lower yields (Table 5, entries 7 and 8). The variation of the amount of PhCN lead to an optimal yield of 83% when 2.5 equiv PhCN were employed (Table 5, entry 9). However, using 3.0 equiv PhCN failed to enhance the yield (Table 5, entry 10).

**Table 5:** The Pd(OAc)_2_-catalyzed carbonylation of tetraphenylene (**1**).

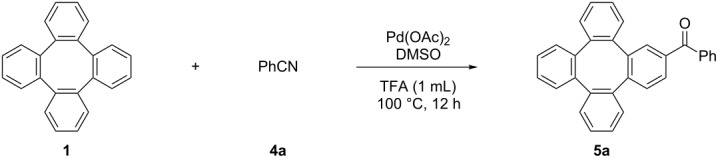					

Entry	PhCN (equiv)	Pd(OAc)_2_ (equiv)	DMSO (equiv)	Additive (0.2 mL)	Yield^a^ (%)

1	2.0	10%	1.0	/	20
2	2.0	10%	2.0	/	42
3	2.0	10%	4.0	/	28
4	2.0	10%	2.0	DCM	52
5	2.0	10%	2.0	DCE	36
6	2.0	20%	2.0	DCM	71
7	2.0	20%	2.0	DCM	60^b^
8	2.0	20%	2.0	DCM	64^c^
**9**	**2.5**	**20%**	**2.0**	**DCM**	**83 (80)**^d^
10	3.0	20%	2.0	DCM	60

^a^The yields were determined by ^1^H NMR analysis of the crude products using CH_2_Br_2_ as the internal standard. ^b^Reaction temperature 90 °C. ^c^Reaction temperature 110 °C. ^d^Isolated yields based on tetraphenylene (**1**).

**Scheme 1:**
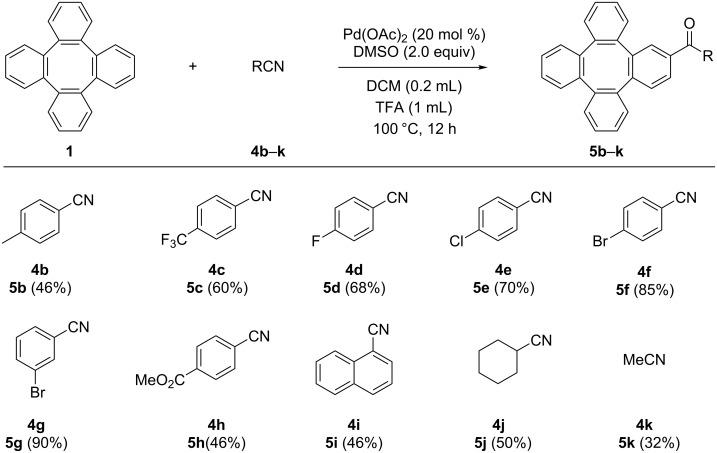
The Pd(OAc)_2_-catalyzed reaction of nitriles with tetraphenylene (**1**).

Under the optimal reaction conditions, various nitriles **4b–k** including aromatic and aliphatic ones, were reacted with tetraphenylene (**1**) to give the corresponding carbonyl products **5b–k** (Scheme 1). Both substrates containing either an electron-donating methyl group or electron-withdrawing trifluoromethyl group were suitable for the reaction. In addition, halogen-substituted nitriles, including F, Cl, and Br substituents, were well-tolerated under the standard reaction conditions. Also, methyl 4-cyanobenzoate and 1-naphthonitrile were successfully reacted with tetraphenylene to form the corresponding carbonylated products. It is worth mentioning that aliphatic nitriles **4j**,**k** were also found to be reactive under the conditions.

## Conclusion

In conclusion, three reactions for halogenation, acetoxylation, and carbonylation of tetraphenylene (**1**) have been developed via a transition-metal-catalyzed direct derivatization. The reactions provide new methods for the synthesis of a variety of 2-substituted tetraphenylenes, which could accelerate the research on the properties and application of tetraphenylene derivatives.

## Supporting Information

File 1 Experimental section and characterization of the synthesized compounds.
