# Supplementary material for: Synthesis of 2-substituted tetraphenylenes via transition-metal-catalyzed derivatization of tetraphenylene
Source: Beilstein J Org Chem. 2016 Jun 22;12:1302–8. doi: 10.3762/bjoc.12.122 (PMC4979733; doi:10.3762/bjoc.12.122)

Supporting Information

for

Synthesis of 2-substituted tetraphenylenes via transition-metal-catalyzed derivatization of tetraphenylene

Shulei Pan, Hang Jiang, Yanghui Zhang*, Yu Zhang, and Dushen Chen

Address: Department of Chemistry, and Shanghai Key Lab of Chemical Assessment and Sustainability, Tongji University, 1239 Siping Road, Shanghai, 200092, P. R. China organization

Email: Yanghui Zhang - zhangyanghui@tongji.edu.cn

*Corresponding author

Experimental section and characterization of the synthesized compounds

General information S2
I. General procedure for screening of reaction conditions S2
II. General procedure for synthesis of 2-substituted tetraphenylenes S4
III. References S6
IV. Characterization of synthesized compounds S6
V. NMR spectra S10

General information: ^{1}H NMR and ^{13}C NMR spectra were recorded with a Bruker ARX400 and a Bruker ARX600. High resolution mass spectra were measured on a Bruker MicroTOF II ESI-TOF mass spectrometer. ^{1}H NMR spectra were recorded in $CDCl_3$ and referenced to residual $CHCl_3$ at 7.26 ppm and ^{13}C NMR spectra were referenced to the central peak of $CDCl_3$ at 77.0 ppm. Multiplicities are reported using the following abbreviations: s = singlet, d = doublet, t = triplet, q = quartet, m = multiplet, br = broad resonance. $Pd(OAc)_2$ was purchased from Strem Chemicals. $AuCl_3$ was purchased from Energy Chemical. The solvents and the inorganic salts were purchased from Tansoole. The solvents were purified by distillation prior to use. Unless otherwise noted, the other commercial materials were used without further purification.

I. General procedure for screening of reaction conditions

1. $Pd(OAc)_2$-catalyzed acetoxylation of tetraphenylene with $PhI(OAc)_2$ [1]

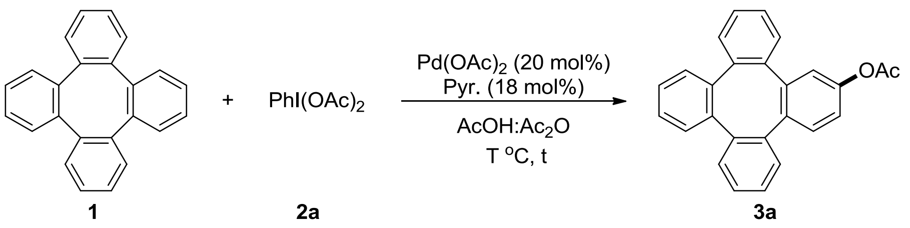

A 25 mL pressure resistant vial equipped with a magnetic stirring bar was charged with $Pd(OAc)_2$ (4.48 mg, 0.02 mmol), followed by pyridine (1.45 μL, 0.018 mmol), $PhI(OAc)_2$ and solvent. The resulting suspension was stirred at room temperature for 1 min, and then tetraphenylene (**1**, 30.4 mg, 0.1 mmol) was added. The vial was sealed with a Teflon-lined cap, and the reaction mixture was heated to the inidacted temperature using a preheated hotplate. After the reaction mixture was stirred for the indicated time, it was allowed to cool to ambient temperature. The reaction mixture was diluted with ethyl acetate and then filtered through a small pad of Celite. The filtrate was concentrated in vacuo. The yield was determined by ^{1}H NMR analysis of crude product using CH_2Br_2 as the internal standard.

2. $AuCl_3$-catalyzed chlorination of tetraphenylene with NCS [2]

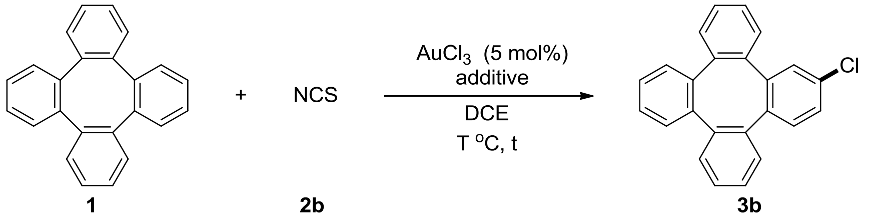

A 25 mL pressure resistant vial equipped with a magnetic stirring bar was charged with tetraphenylene (**1**, 30.4 mg, 0.10 mmol), followed by $AuCl_3$ (1.5 mg, 0.005 mmol), NCS and DCE (1 mL). The resulting suspension was stirred at room temperature for 1 min, and then the additive was added. After the resulting reaction mixture was allowed to stir for the indicated time at the indicated temperature, it was allowed to cool to ambient temperature. The reaction mixture was diluted with ethyl acetate and then filtered through a small pad of Celite. The filtrate was

concentrated in vacuo. The yield was determined by ^{1}H NMR analysis of crude product using CH_2Br_2 as the internal standard.

3. $AuCl_3$-catalyzed bromination of tetraphenylene with NBS [2]

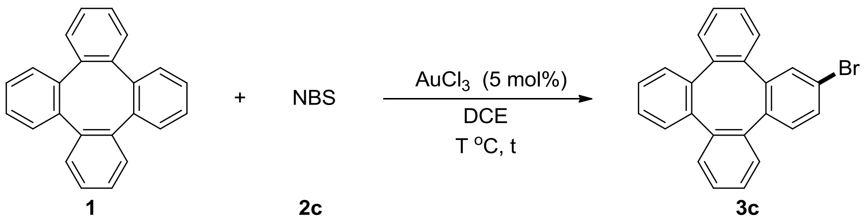

A 25 mL pressure resistant vial equipped with a magnetic stirring bar was charged with tetraphenylene (**1**, 30.4 mg, 0.10 mmol), followed by $AuCl_3$ (1.5 mg, 0.005 mmol), NBS and DCE (1 mL). After the resulting reaction mixture was allowed to stir for the indicated time at the indicated temperature it was allowed to cool to ambient temperature. The reaction mixture was diluted with ethyl acetate and then filtered through a small pad of Celite. The filtrate was concentrated in vacuo. The yield was determined by ^{1}H NMR analysis of crude product using CH_2Br_2 as the internal standard.

4. $AuCl_3$-catalyzed iodination of tetraphenylene with NIS [2]

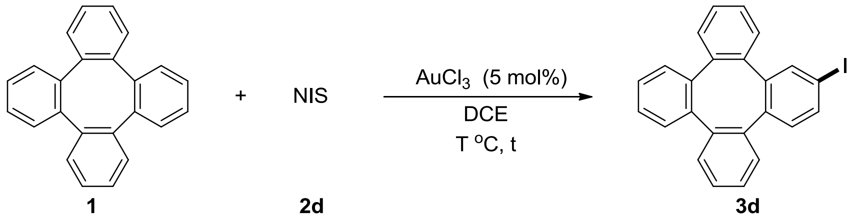

A 25 mL pressure resistant vial equipped with a magnetic stirring bar was charged with tetraphenylene (**1**, 30.4 mg, 0.10 mmol), followed by $AuCl_3$ (1.5 mg, 0.005 mmol), NIS and DCE (1 mL). After the resulting reaction mixture was allowed to stir for the indicated time at the indicated temperature, it was allowed to cool to ambient temperature. The reaction mixture was diluted with ethyl acetate and then filtered through a small pad of Celite. The filtrate was concentrated in vacuo. The yield was determined by ^{1}H NMR analysis of crude product using CH_2Br_2 as the internal standard.

5. $Pd(OAc)_2$-catalyzed carbonylation of tetraphenylene with nitriles [3]

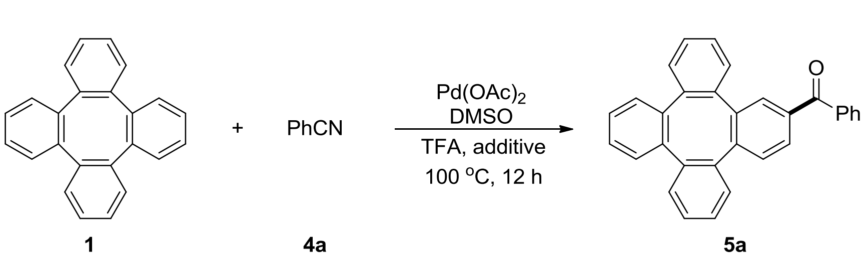

A 25 mL pressure resistant vial equipped with a magnetic stirring bar was charged with tetraphenylene (**1**, 30.4 mg, 0.10 mmol), followed by $Pd(OAc)_2$, PhCN, DMSO, additive and

trifluoroacetic acid (1 mL). The vial was sealed with a Teflon-lined cap, and the resulting reaction mixture was allowed to stir for 12 h at 100 °C. After cooling to room temperature, water (5 mL) was added to the mixture and the resulting mixture was heated at 120 °C for 2 h. After cooling to room temperature, the reaction mixture was diluted with ethyl acetate (20 mL), and then filtered through a pad of Celite. The filtrate was washed with brine (10 mL), dried over Na_2SO_4, and concentrated in vacuo. The yield was determined by ^1H NMR analysis of crude product using CH_2Br_2 as the internal standard.

II. General procedure for synthesis of 2-substituted tetraphenylenes

1. Synthesis of 2-acetoxytetraphenylene

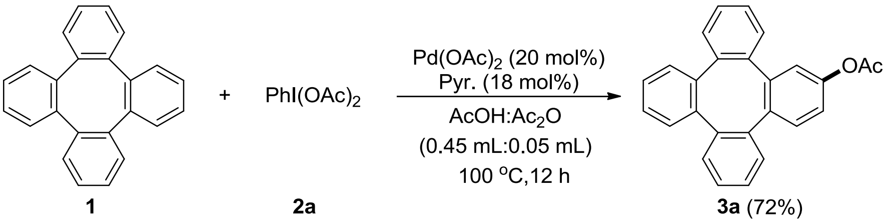

A solution of $PhI(OAc)_2$ (96.6 mg, 0.30 mmol), $Pd(OAc)_2$ (4.5 mg, 0.02 mmol), pyridine (1.4 µL, 0.018 mmol) and Ac_2O (0.05 mL) in AcOH (0.45 mL) was stirred at for 1min at room temperature. Then tetraphenylene (**1**, 30.4 mg, 0.10 mmol) was added and the reaction mixture was stirred for 12 h at 100 °C. At the end of the reaction, the solution was diluted with ethyl acetate (10 mL) and then filtered through a pad of Celite. The filtrate was concentrated in vacuo and the crude product was purified by preparative silica gel chromatography (eluent: petroleum ether/EtOAc 10:1).

2. Synthesis of 2-chlorotetraphenylene

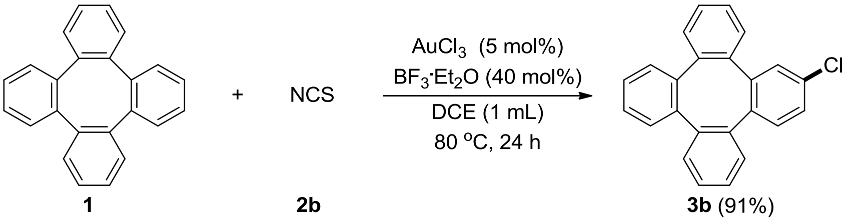

A solution of tetraphenylene (**1**, 30.4 mg, 0.10 mmol), NCS (26.7 mg, 0.20 mmol) and $AuCl_3$ (1.5 mg, 0.005 mmol) in DCE (1 mL) was stirred at room temperature for 1 min, and then $BF_3{\cdot}Et_2O$ (4.9 µL, 0.04 mmol) was added. The resulting reaction mixture was allowed to stir at 80 °C for 24 h. At the end of the reaction, the solution was diluted with ethyl acetate (10 mL) and then filtered through a pad of Celite. The filtrate was concentrated in vacuo and the crude product was purified by preparative silica gel chromatography (eluent: petroleum ether).

3. Synthesis of 2-bromotetraphenylene

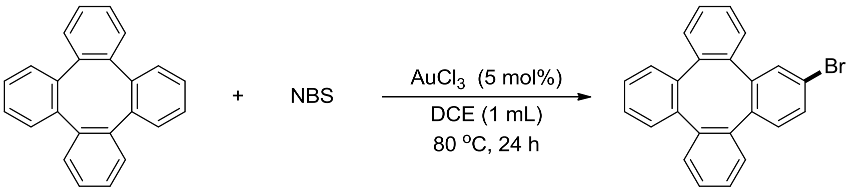

1 **2c** **3c** (95%)

A solution of tetraphenylene (**1**, 30.4 mg, 0.10 mmol), NBS (26.7 mg, 0.15 mmol) and $AuCl_3$ (1.5 mg, 0.005 mmol) in DCE (1 mL) was stirred at 80 °C for 24 h. At the end of the reaction, the solution was diluted with ethyl acetate (10 mL) and then filtered through a pad of Celite. The filtrate was concentrated in vacuo and the crude product was purified by preparative silica gel chromatography (eluent: petroleum ether).

4. Synthesis of 2-iodotetraphenylene

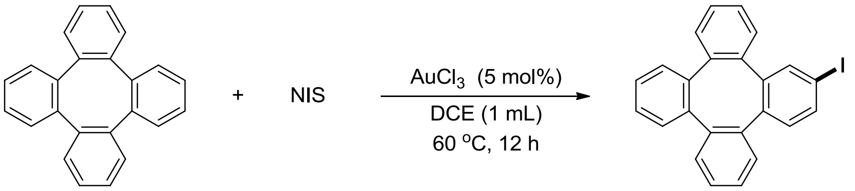

1 **2d** **3d** (78%)

A solution of compound **1** (30.4 mg, 0.10 mmol), NIS (45.0 mg, 0.20 mmol) and $AuCl_3$ (1.5 mg, 0.005 mmol) in DCE (1 mL) was stirred at 60 °C for 12 h. At the end of the reaction, the solution was diluted with ethyl acetate (10 mL) and then filtered through a pad of Celite. The filtrate was concentrated in vacuo and the crude product was purified by preparative silica gel chromatography (eluent: petroleum ether).

5. Synthesis of 2-carbonyltetraphenylenes

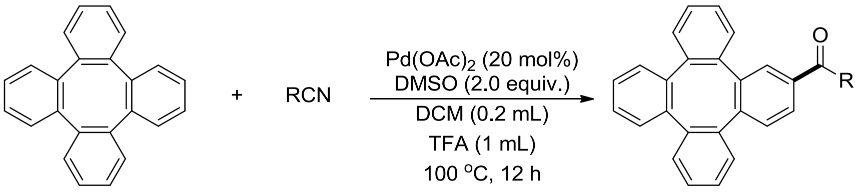

1 **4a-k** **5a-k** (32-90%)

A 25 mL pressure resistant vial equipped with a magnetic stir bar was charged with tetraphenylene (**1**, 30.4 mg, 0.10 mmol), followed by $Pd(OAc)_2$ (4.5 mg, 0.02 mmol), DMSO (14.2 µL, 0.2 mmol), RCN (0.25 mmol), DCM (0.2 mL) and trifluoroacetic acid (1 mL). The vial was sealed with a Teflon-lined cap, and the resulting reaction mixture was allowed to stir for 12 h at 100 °C. After cooling to room temperature, water (5 mL) was added to the mixture and the resulting mixture was heated at 120 °C for 2 h. After cooling to room temperature, the reaction mixture was diluted with ethyl acetate (20 mL), and then filtered through a pad of Celite. The filtrate was washed with brine (10 mL), dried over Na_2SO_4, and concentrated in vacuo. The crude product was purified by preparative silica gel chromatography (eluent: petroleum ether/EtOAc 10:1).

III. References

1. Emmert, M. H.; Cook, A. K.; Xie, Y. J.; Sanford, M. S. *Angew. Chem. Int. Ed.*, **2011**, *50*, 9409-9412.
2. Mo, F.; Yan, J. M.; Qiu, D.; Li, F.; Zhang, Y.; Wang, J. *Angew. Chem. Int. Ed.,* **2010**, *49*, 2028-2032.
3. Zhou, C.; Larock, R. C. *J. Am. Chem Soc.*, **2004**, *126*, 2302-2303.

IV. Characterization of synthesized compounds

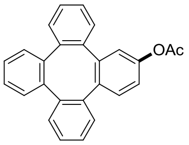

3a

White solid, m.p.:198 ^{o}C
^{1}H NMR (400 MHz, CDCl$_3$) δ 7.32 – 7.26 (m, 6H), 7.18 – 7.16 (m, 7H), 7.03 (dd, *J* = 8.3, 2.4 Hz, 1H), 6.91 (d, *J* = 2.3 Hz, 1H), 2.27 (s, 3H); **^{13}C NMR (101 MHz, CDCl$_3$)** δ 169.36, 149.63, 142.79, 141.57, 141.41, 141.33, 141.25, 140.64, 140.54, 139.15, 130.08, 129.13, 129.08, 129.07, 128.98, 127.53, 127.38, 127.35, 127.32, 127.27, 121.81, 120.25, 21.16. **HRMS (ESI-TOF) *m/z***: calcd for $C_{26}H_{18}ONa^{+}$: 385.1199 (M + Na)$^{+}$, found: 385.1205.

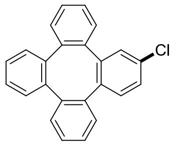

3b

White solid, m.p.:163 ^{o}C
^{1}H NMR (600 MHz, CDCl$_3$) δ 7.32 – 7.27 (m, 6H), 7.25 (dd, *J* = 8.3, 2.3 Hz, 1H), 7.19 – 7.12 (m, 7H), 7.10 (d, *J* = 8.2 Hz, 1H); **^{13}C NMR (101 MHz, CDCl$_3$)** δ 143.10, 141.46, 141.38, 141.18, 141.11, 140.30, 140.15, 140.05, 132.90, 130.32, 129.13, 129.05, 128.89, 128.80, 127.70, 127.54, 127.38, 127.33. **HRMS (ESI-TOF) *m/z***: calcd for $C_{24}H_{15}ClNa^{+}$: 361.0754 (M + Na)$^{+}$, found: 361.0751.

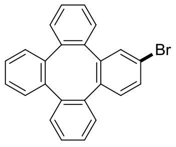

3c

White solid, m.p.:172 ^{o}C
^{1}H NMR (400 MHz, CDCl$_3$) δ 7.40 (dd, *J* = 8.2, 2.0 Hz, 1H), 7.33 (d, *J* = 2.0 Hz, 1H), 7.32 – 7.27 (m, 6H), 7.20 – 7.10 (m, 6H), 7.03 (d, *J* = 8.2 Hz, 1H); **^{13}C NMR (101 MHz, CDCl$_3$)** δ 143.44, 141.42, 141.18, 141.11, 140.55, 140.32, 140.05, 131.75, 130.59, 130.27, 129.15, 129.05, 128.82, 128.81, 127.72, 127.56, 127.40, 127.33, 121.11. **HRMS (ESI-TOF) *m/z***: calcd for $C_{24}H_{15}BrNa^{+}$: 405.0249 (M + Na)$^{+}$, found: 405.0247.

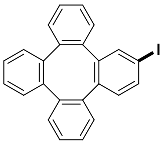

3d

White solid, m.p.:178 ^{o}C
^{1}H NMR (400 MHz, CDCl$_3$) δ 7.64 (dd, *J* = 8.1, 1.7 Hz, 1H), 7.57 (d, *J* = 1.7 Hz, 1H), 7.38 – 7.31 (m, 6H), 7.24 – 7.14 (m, 6H), 6.94 (d, *J* = 8.1 Hz, 1H); **^{13}C NMR (101 MHz, CDCl$_3$)** δ 143.62, 141.40, 141.39, 141.19, 141.14, 140.40, 139.91, 137.62, 136.24, 130.76, 130.32, 129.17, 129.14, 129.07, 128.90, 128.88, 128.86, 128.81, 128.77, 127.70, 127.57, 127.41, 127.34, 92.91. **HRMS (ESI-TOF) *m/z***: calcd for $C_{24}H_{15}INa^{+}$: 453.0111 (M + Na)$^{+}$, found: 453.0102.

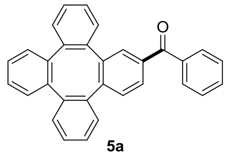

5a

White solid, m.p.:151 °C
^{1}H NMR (400 MHz, $CDCl_3$) δ 7.80 (d, *J* = 7.1 Hz, 2H), 7.74 (dd, *J* = 7.9, 1.7 Hz, 1H), 7.65 (d, *J* = 1.6 Hz, 1H), 7.57 (t, *J* = 7.4 Hz, 1H), 7.47 (t, *J* = 7.6 Hz, 2H), 7.33 – 7.28 (m, 7H), 7.23 – 7.14 (m, 6H); **^{13}C NMR (101 MHz, $CDCl_3$)** δ 196.30, 145.95, 141.77, 141.48, 141.37, 141.14, 141.07, 140.57, 140.48, 137.63, 136.35, 132.29, 130.81, 129.95, 129.23, 129.17, 129.12, 129.07, 129.04, 128.72, 128.25, 127.81, 127.67, 127.48, 127.41, 127.36. **HRMS (ESI-TOF) *m/z***: calcd for $C_{31}H_{20}OK^+$: 447.1146 $(M + K)^+$, found: 447.1170.

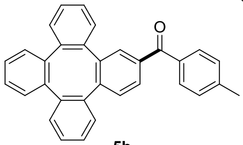

5b

Amorphous
^{1}H NMR (400 MHz, $CDCl_3$) δ 7.75 – 7.69 (m, 3H), 7.62 (d, *J* = 1.6 Hz, 1H), 7.33 – 7.28 (m, 9H), 7.23 – 7.14 (m, 6H), 2.43 (s, 3H); **^{13}C NMR (101 MHz, $CDCl_3$)** δ 196.06, 145.68, 143.10, 141.66, 141.48, 141.3, 141.16, 141.09, 140.62, 140.54, 136.68, 134.91, 130.75, 130.20, 129.21, 129.17, 129.12, 129.04, 128.93, 128.78, 128.74, 127.77, 127.62, 127.46, 127.39, 127.38, 127.34, 21.61. **HRMS (ESI-TOF) *m/z***: calcd for $C_{32}H_{22}ONa^+$: 445.1563 $(M + Na)^+$, found: 445.1565.

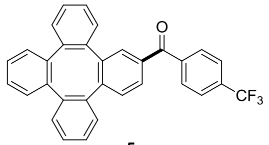

5c

Amorphous
^{1}H NMR (400 MHz, $CDCl_3$) δ 7.88 (d, *J* = 8.1 Hz, 2H), 7.75 – 7.73 (m, 3H), 7.64 (d, *J* = 1.6 Hz, 1H), 7.33 – 7.30 (m, 7H), 7.21 – 7.14 (m, 6H); **^{13}C NMR (101 MHz, $CDCl_3$)** δ 195.07, 146.70, 142.10, 141.47, 141.33, 141.07, 141.01, 140.78 (q, J_{C-F} = 0.8 Hz) 140.35, 140.24, 135.50, 133.61 (q, J_{C-F} = 32.2 Hz) 130.82, 130.02, 129.42, 129.29, 129.22, 129.17, 129.09, 128.96, 128.65, 127.96, 127.83, 127.53, 127.47, 127.42, 125.33 (q, J_{C-F} = 3.7 Hz), 123.64 (q, J_{C-F} = 270.93 Hz). **HRMS (ESI-TOF) *m/z***: calcd for $C_{32}H_{19}F_3ONa^+$: 499.1280 $(M + Na)^+$, found: 499.1280.

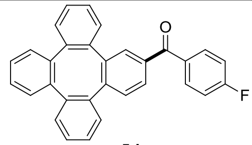

5d

Amorphous
^{1}H NMR (400 MHz, $CDCl_3$) δ 7.87 – 7.80 (m, 2H), 7.70 (dd, *J* = 7.9, 1.8 Hz, 1H), 7.60 (d, *J* = 1.6 Hz, 1H), 7.33 – 7.28 (m, 7H), 7.20 – 7.14 (m, 8H); **^{13}C NMR (101 MHz, $CDCl_3$)** δ 194.86, 165.29 (d, J_{C-F} = 253.9 Hz), 146.03, 141.85, 141.45, 141.32, 141.09, 141.03, 140.46, 140.36, 136.23, 133.78 (d, J_{C-F} = 3.0 Hz), 132.57 (d, J_{C-F} = 9.0 Hz), 130.67, 129.25, 129.20, 129.15, 129.00, 128.91, 128.71, 127.86, 127.74, 127.49, 127.43, 127.38, 115.44 (d, J_{C-F} = 21.8 Hz), 29.68. **HRMS (ESI-TOF) *m/z***: calcd for $C_{31}H_{19}FONa^+$: 449.1312 $(M + Na)^+$, found: 449.1317.

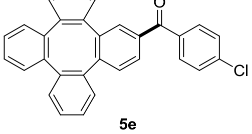

5e

White solid, m.p.:170 °C
^{1}H NMR (400 MHz, $CDCl_3$) δ 7.74 (d, *J* = 8.5 Hz, 2H), 7.70 (dd, *J* = 7.9, 1.7 Hz, 1H), 7.61 (d, *J* = 1.6 Hz, 1H), 7.44 (d, *J* = 8.5 Hz, 2H), 7.32

– 7.29 (m, 7H), 7.19 – 7.16(m, 6H); **^{13}C NMR (101 MHz, CDCl$_3$)** δ 195.04, 146.22, 141.93, 141.46, 141.34, 141.10, 141.04, 140.44, 140.33, 138.77, 136.00, 135.90, 131.35, 130.70, 129.28, 129.25, 129.19, 129.17, 129.14, 128.98, 128.92, 128.69, 128.60, 127.88, 127.75, 127.49, 127.44, 127.38. **HRMS (ESI-TOF) *m/z***: calcd for $C_{31}H_{19}ClONa^+$:465.1017 $(M + Na)^+$, found: 465.1021.

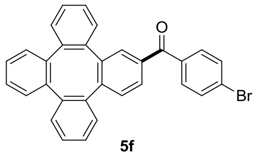

5f

White solid, m.p.:176 ^oC

^{1}H NMR (400 MHz, CDCl$_3$) δ 7.70 (dd, *J* = 7.9, 1.8 Hz, 1H), 7.68 – 7.58 (m, 5H), 7.35 – 7.27 (m, 7H), 7.22 – 7.14 (m, 6H); **^{13}C NMR (101 MHz, CDCl$_3$)** δ 195.18, 146.26, 141.94, 141.46, 141.34, 141.10, 141.04, 140.44, 140.33, 136.36, 135.93, 131.58, 131.46, 130.72, 129.30, 129.26, 129.19, 129.18, 129.15, 128.99, 128.94, 128.69, 127.89, 127.76, 127.50, 127.44, 127.39. **HRMS (ESI-TOF) *m/z***: calcd for $C_{31}H_{19}BrONa^+$: 509.0511 $(M + Na)^+$, found: 509.0522.

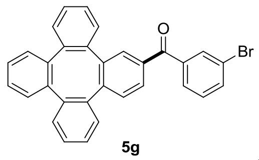

5g

White solid, m.p.:200 ^oC

^{1}H NMR (400 MHz, CDCl$_3$) δ 7.92 (s, 1H), 7.73 – 7.66 (m, 3H), 7.63 (d, *J* = 1.5 Hz, 1H), 7.38 – 7.27 (m, 8H), 7.22 – 7.12 (m, 6H); **^{13}C NMR (101 MHz, CDCl$_3$)** δ 194.67, 146.42, 142.02, 141.49, 141.36, 141.09, 141.02, 140.43, 140.32, 139.52, 135.68, 135.14, 132.74, 130.75, 129.84, 129.31, 129.26, 129.16, 129.14, 129.01, 128.99, 128.67, 128.41, 127.90, 127.77, 127.53, 127.45, 127.39, 122.55. **HRMS (ESI-TOF) *m/z***: calcd for $C_{31}H_{19}BrONa^+$: 509.0511 $(M + Na)^+$, found: 509.0521.

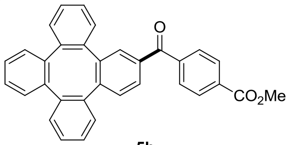

5h

Amorphous

^{1}H NMR (400 MHz, CDCl$_3$) δ 8.13 (d, *J* = 8.5 Hz, 2H), 7.83 (d, *J* = 8.5 Hz, 2H), 7.73 (dd, *J* = 7.9, 1.8 Hz, 1H), 7.63 (d, *J* = 1.6 Hz, 1H), 7.36 – 7.27 (m, 7H), 7.22 – 7.12 (m, 6H), 3.95 (s, 3H); **^{13}C NMR (100 MHz, CDCl$_3$)** δ 195.59, 166.29, 146.52, 142.00, 141.45, 141.39, 141.34, 141.08, 141.01, 140.41, 140.29, 135.71, 133.10, 130.86, 129.67, 129.47, 129.35, 129.27, 129.18, 129.15, 129.08, 128.98, 128.67, 127.92, 127.78, 127.51, 127.45, 127.40, 52.43. **HRMS (ESI-TOF) *m/z***: calcd for $C_{33}H_{22}O_3Na^+$: 489.1461 $(M + Na)^+$, found: 489.1465.

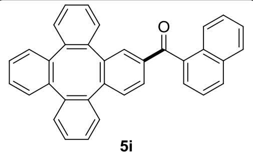

5i

White solid, m.p.:200 ^oC

^{1}H NMR (400 MHz, CDCl$_3$) δ 8.09 (d, *J* = 8.0 Hz, 1H), 7.98 (d, *J* = 8.1 Hz, 1H), 7.90 (d, *J* = 7.4 Hz, 1H), 7.77 (dd, *J* = 7.9, 1.7 Hz, 1H), 7.71 (d, *J* = 1.6 Hz, 1H), 7.60 (d, *J* = 6.3 Hz, 1H), 7.53 – 7.48 (m, 3H), 7.33 – 7.25 (m, 7H), 7.20 – 7.12 (m, 6H); **^{13}C NMR (101 MHz, CDCl$_3$)** δ 197.45, 146.65, 141.90, 141.36, 141.28, 141.07, 140.99, 140.47, 140.34, 137.08, 136.12, 133.66, 131.26, 131.04, 130.89, 129.40, 129.33, 129.24, 129.22, 129.12, 129.05, 128.67, 128.33, 127.86, 127.81, 127.66, 127.44, 127.41, 127.38, 127.35, 127.20, 126.40, 125.64, 124.25. **HRMS (ESI-TOF) *m/z***: calcd for $C_{35}H_{22}ONa^+$: 481.1563 $(M + Na)^+$, found: 481.1565.

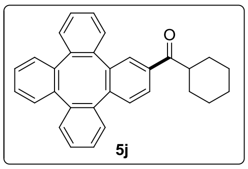

Amorphous

^{1}H NMR (400 MHz, CDCl$_3$) δ 7.91 (dd, *J* = 8.0, 1.7 Hz, 1H), 7.78 (d, *J* = 1.5 Hz, 1H), 7.37 – 7.29 (m, 7H), 7.27 – 7.15 (m, 6H), 3.42 – 3.07 (m, 1H), 1.99 – 1.82 (m, 4H), 1.40 – 1.30 (m, 6H); **^{13}C NMR (101 MHz, CDCl$_3$)** δ 203.41, 146.14, 142.00, 141.51, 141.36, 141.15, 141.11, 140.71, 140.63, 135.28, 129.46, 129.21, 129.17, 129.09, 128.97, 128.67, 127.77, 127.65, 127.43, 127.40, 127.34, 127.19, 45.58, 29.40, 25.94, 25.82. **HRMS (ESI-TOF) *m/z***: calcd for $C_{31}H_{26}ONa^{+}$: 437.1876 (M + Na)$^{+}$, found: 437.1879.

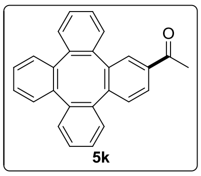

White solid, m.p.:228 °C

^{1}H NMR (400 MHz, CDCl$_3$) δ 7.87 (dd, *J* = 8.0, 1.6 Hz, 1H), 7.76 (d, *J* = 1.3 Hz, 1H), 7.35 – 7.27 (m, 7H), 7.21 – 7.11 (m, 6H), 2.57 (s, 3H); **^{13}C NMR (101 MHz, CDCl$_3$)** δ 197.83, 146.47, 141.96, 141.42, 141.26, 141.05, 141.00, 140.48, 135.98, 129.44, 129.21, 129.15, 129.13, 129.12, 129.10, 129.00, 128.60, 127.83, 127.71, 127.44, 127.41, 127.36, 127.17, 26.66. **HRMS (ESI-TOF) *m/z***: calcd for $C_{26}H_{18}ONa^{+}$: 347.1430 (M + Na)$^{+}$, found: 347.1412.

V. NMR spectra

^{1}H NMR-3a

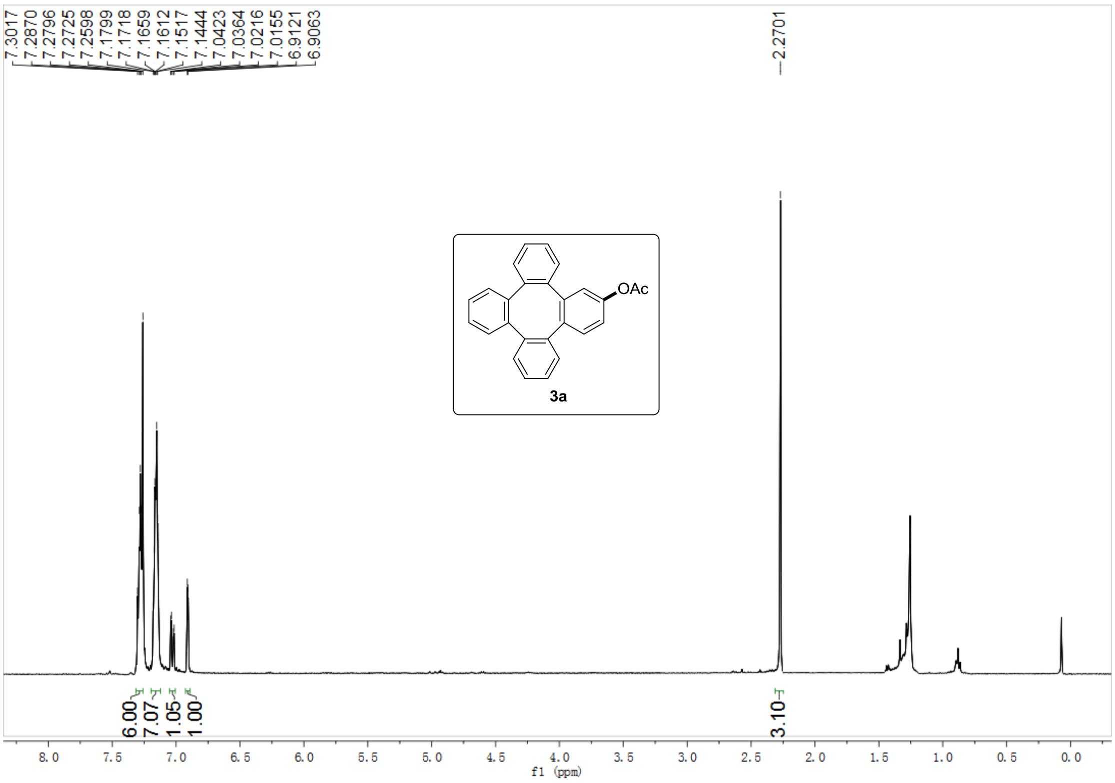

^{13}C NMR-3a

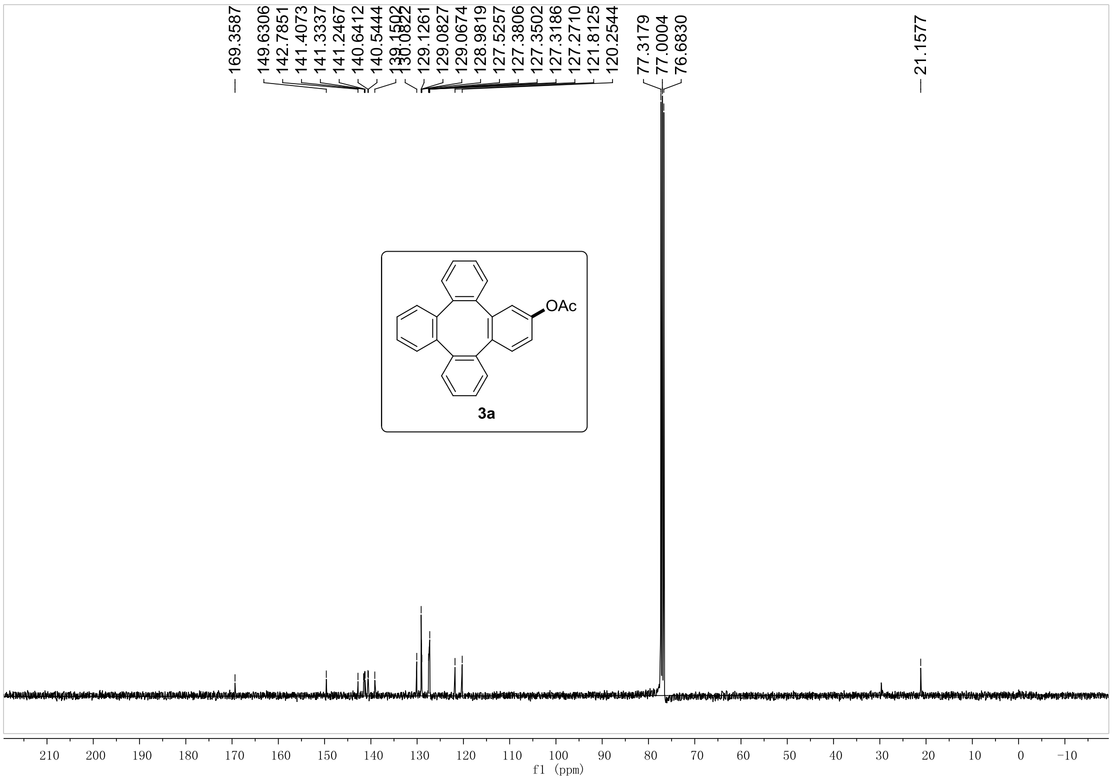

1H NMR-3b

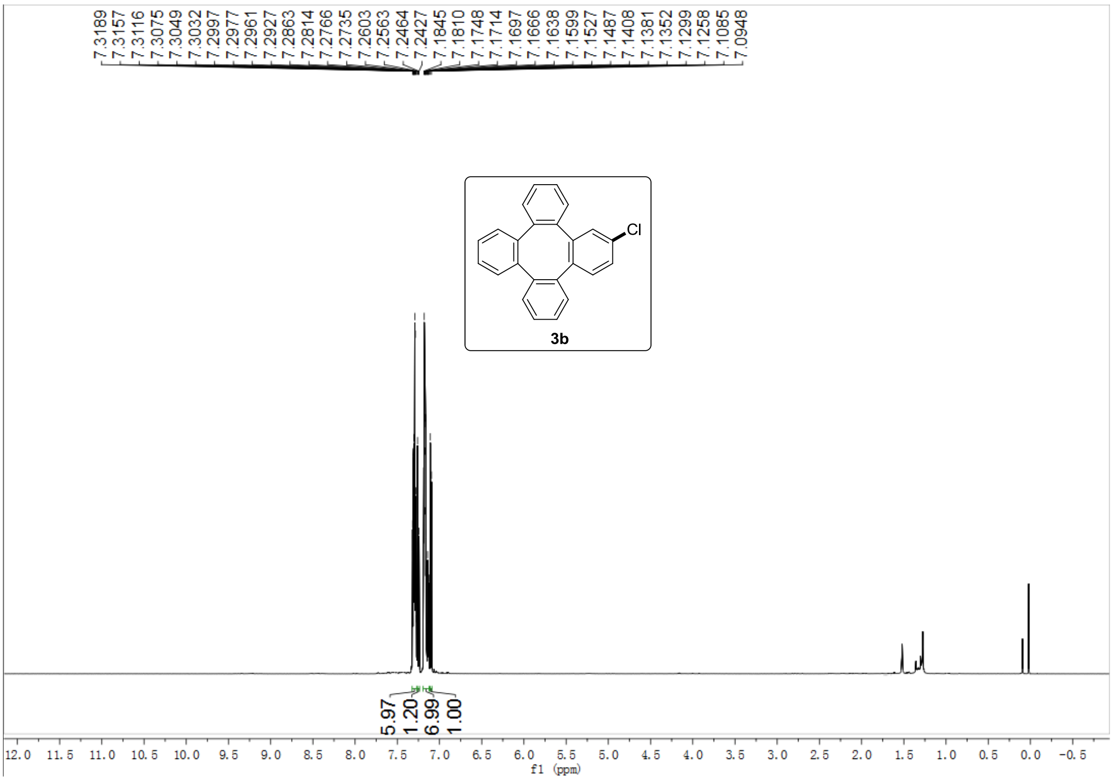

13C NMR-3b

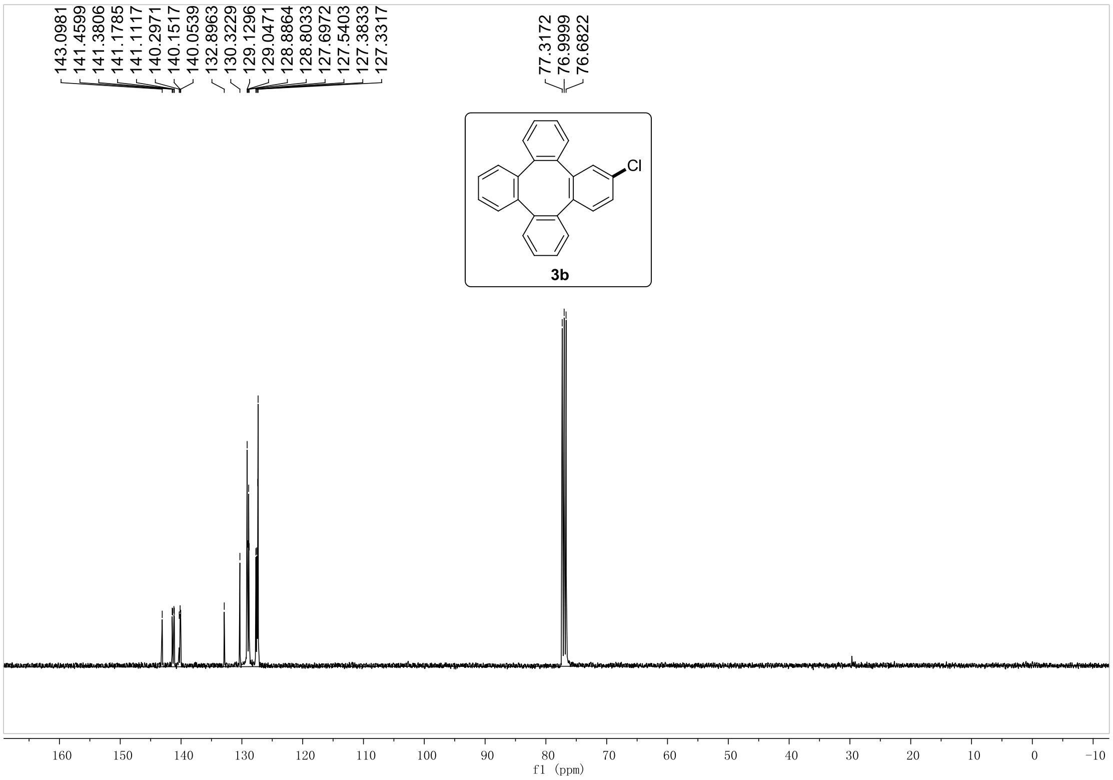

^{1}H NMR-3c

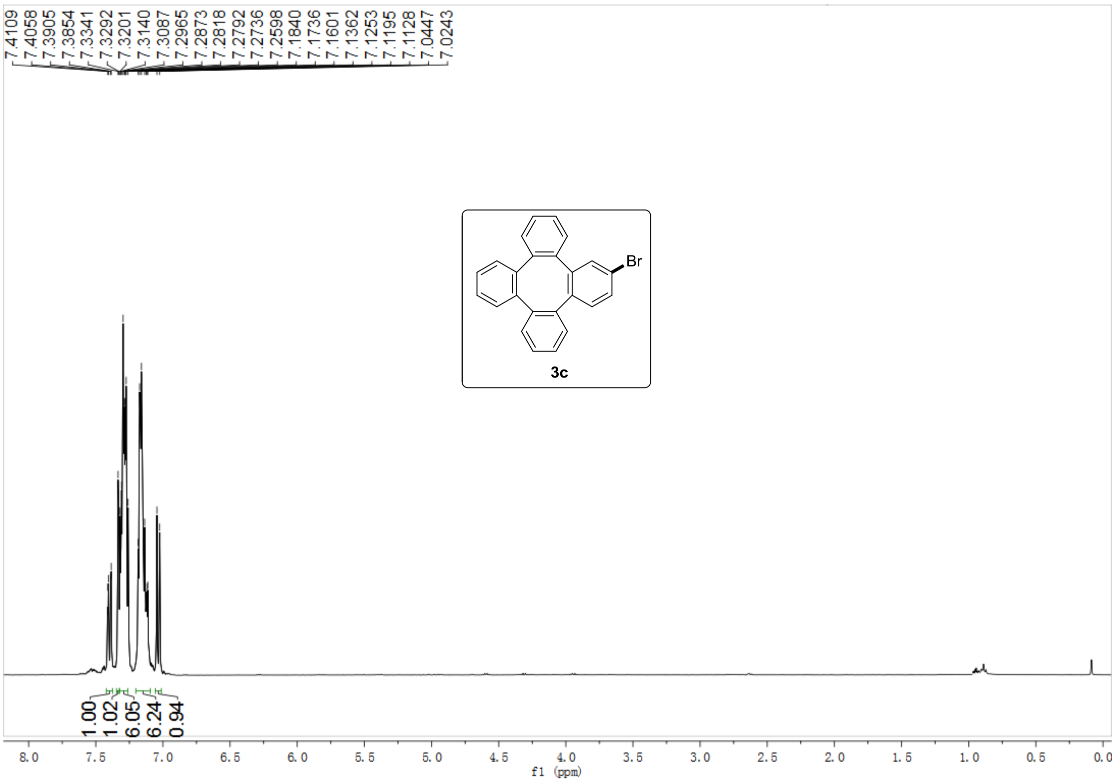

^{13}C NMR-3c

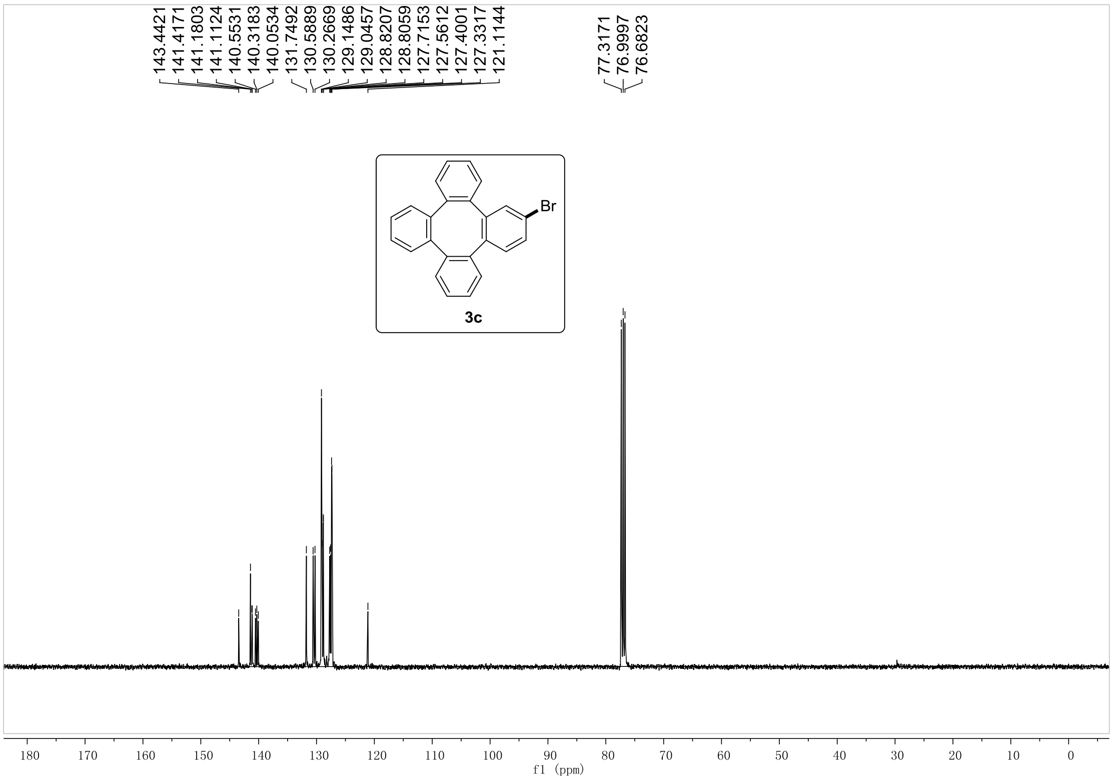

^{1}H NMR-3d

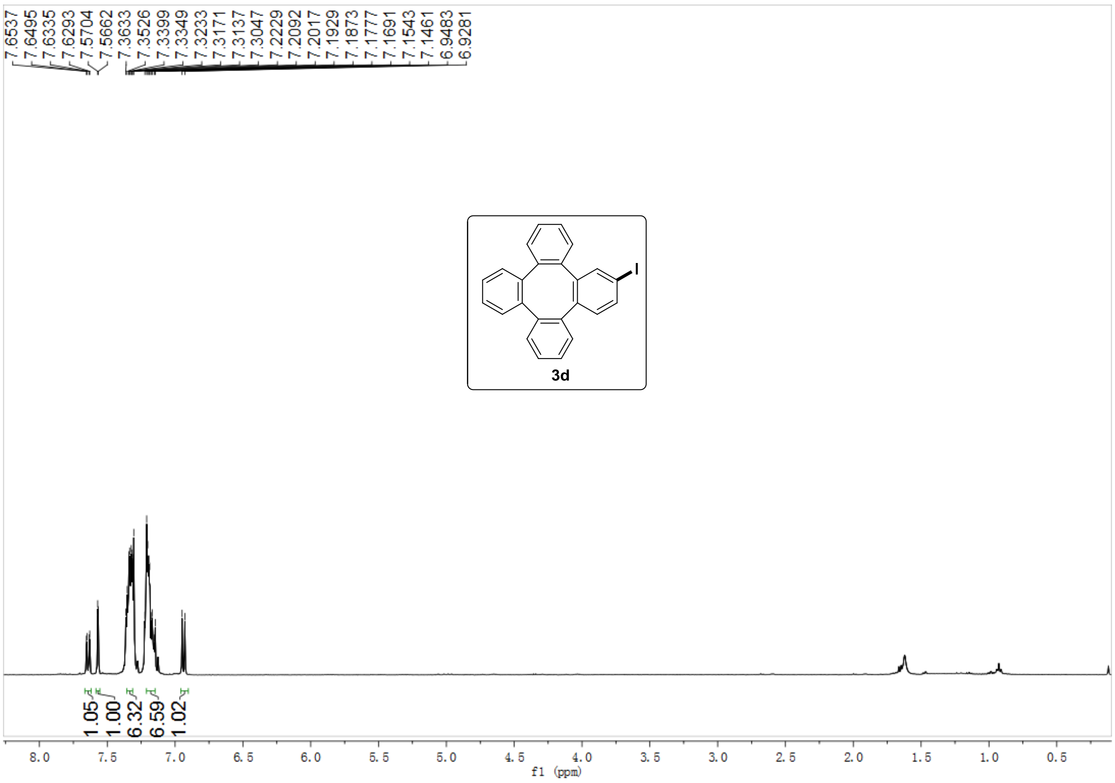

^{13}C NMR-3d

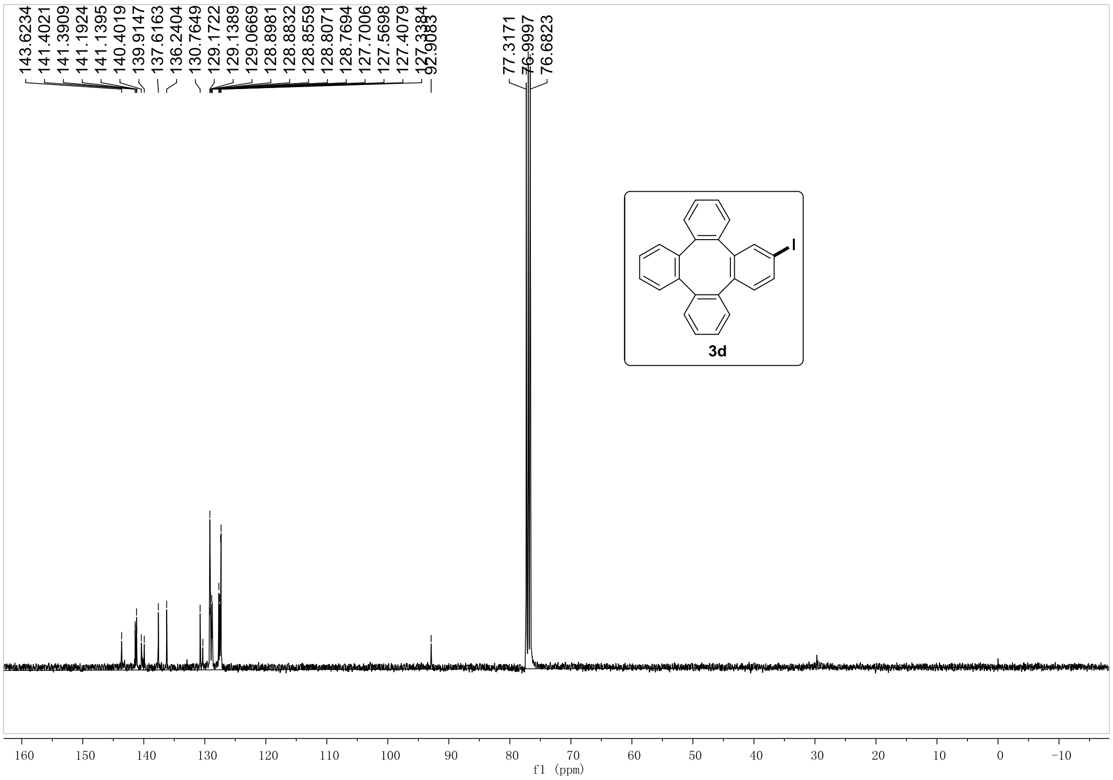

^{1}H NMR-5a

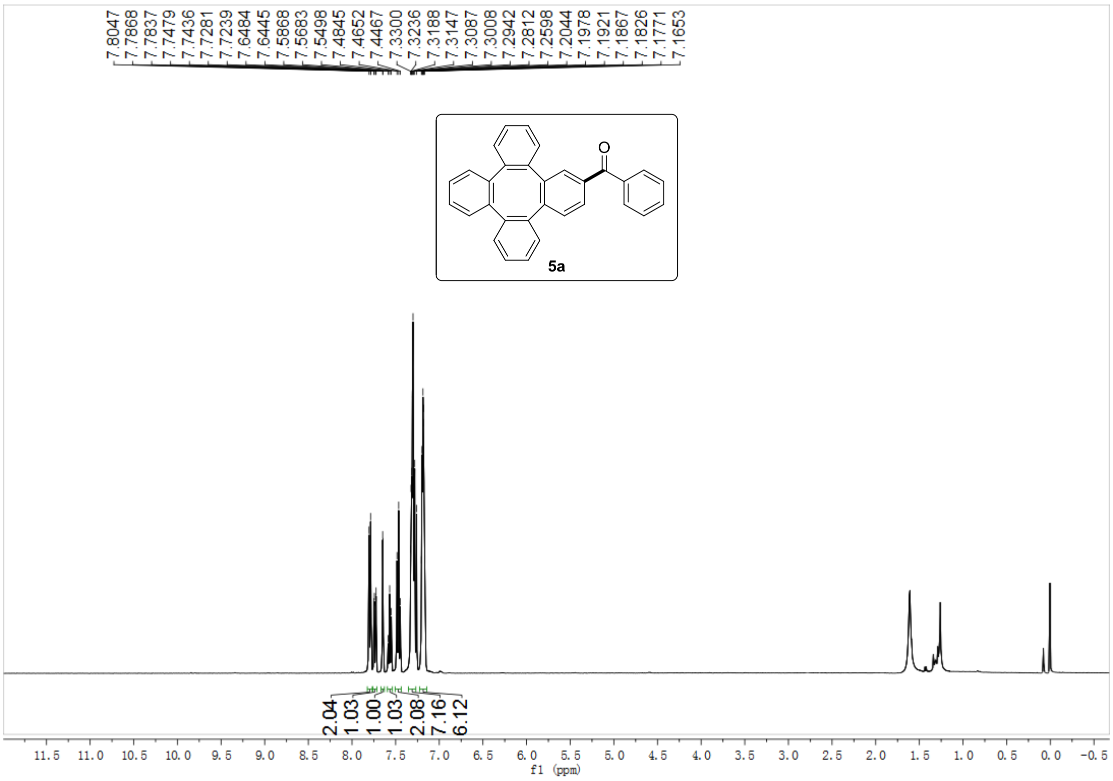

^{13}C NMR-5a

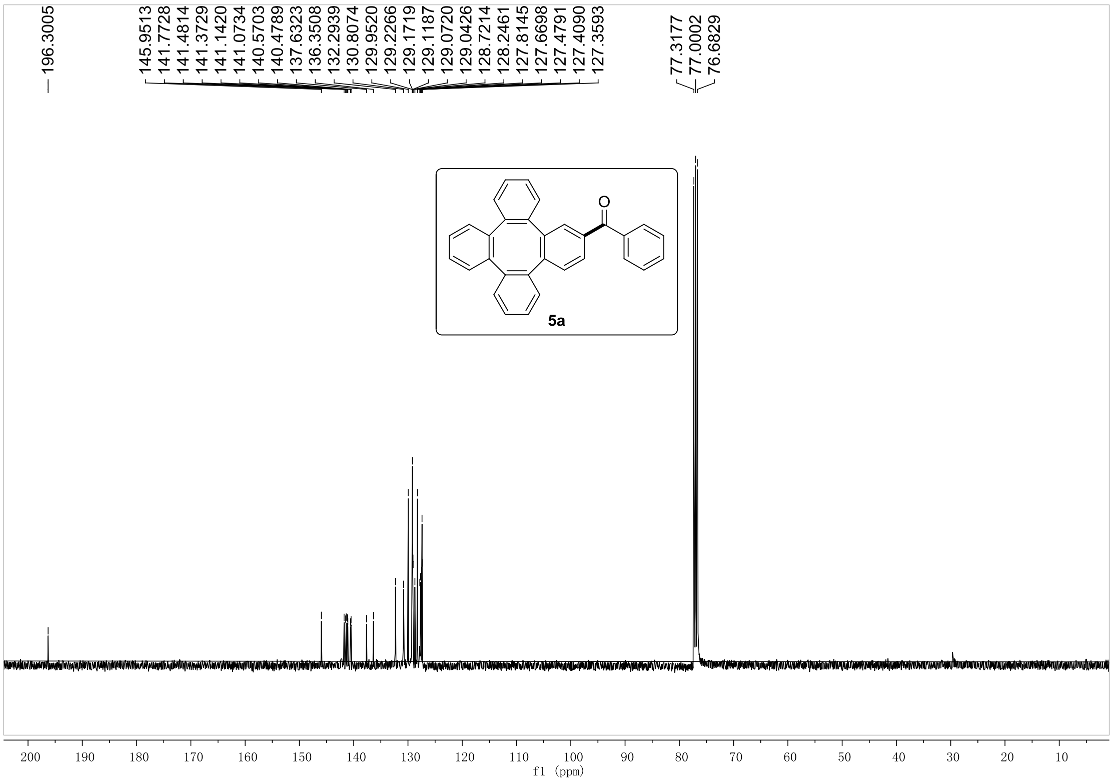

^{1}H NMR-5b

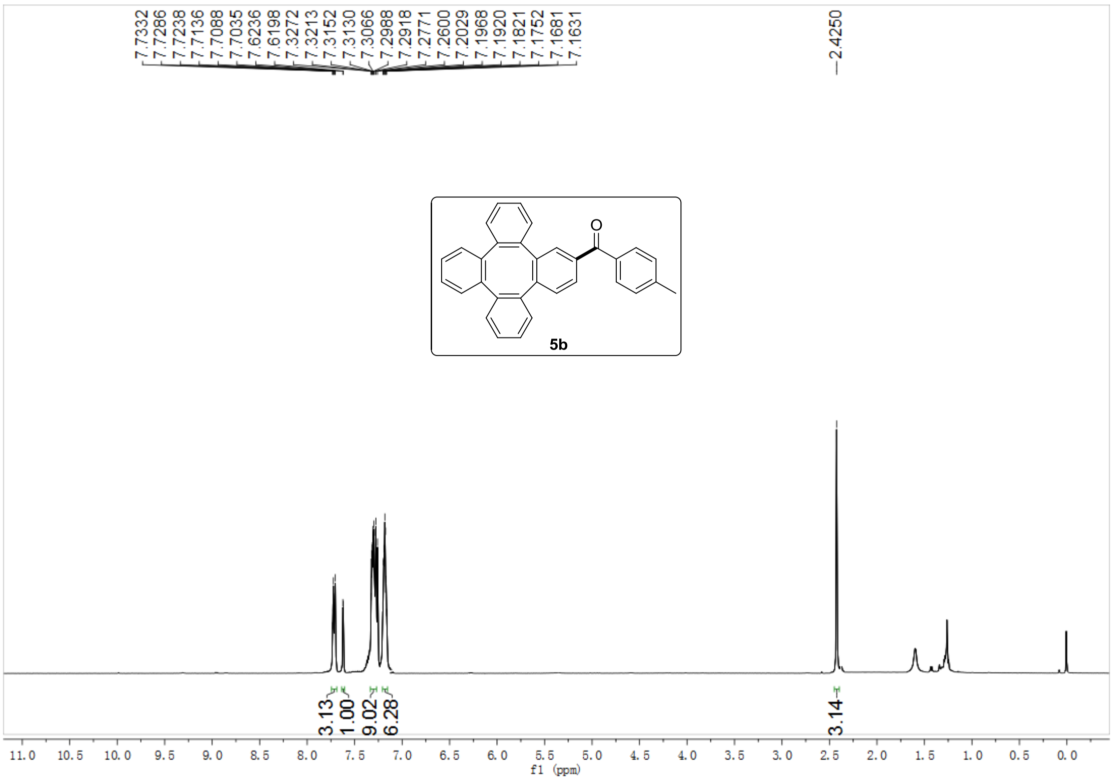

^{13}C NMR-5b

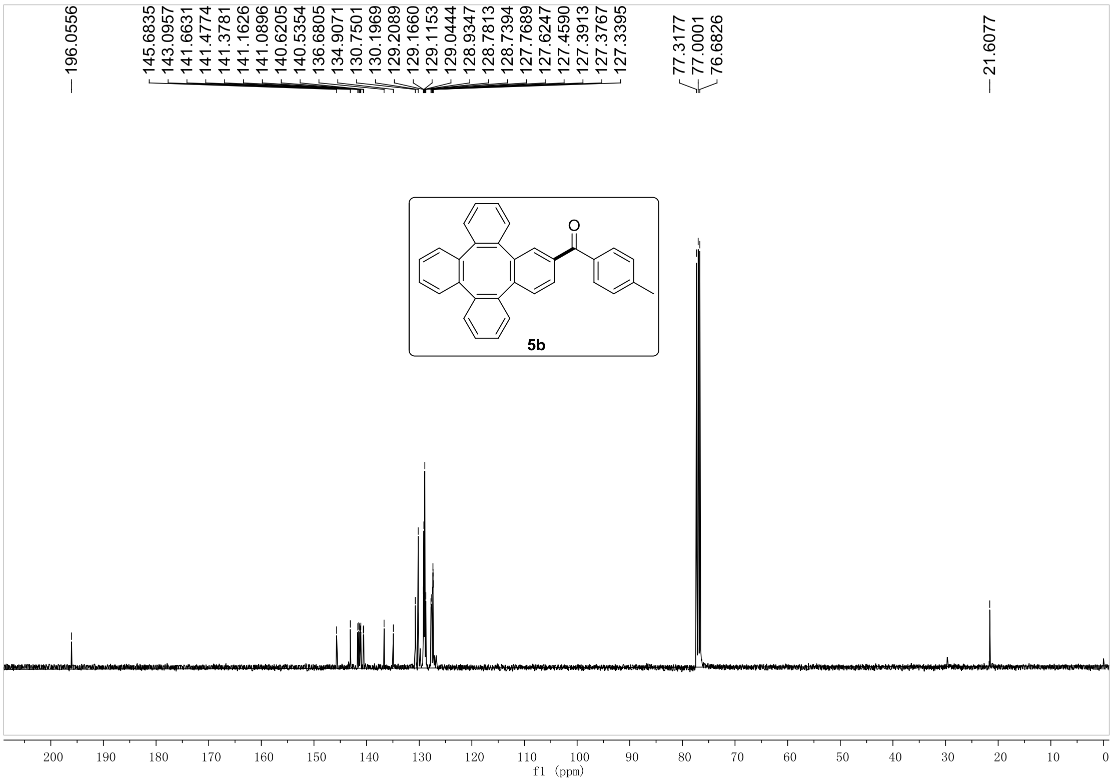

^{1}H NMR-5c

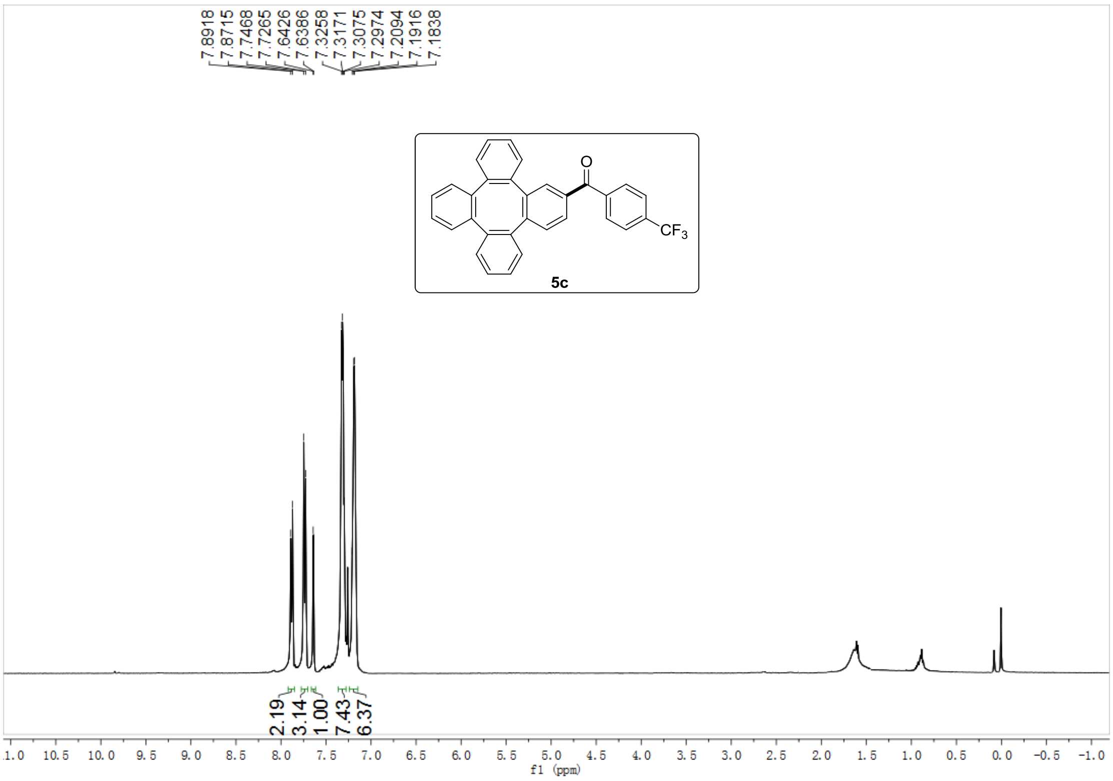

^{13}C NMR-5c

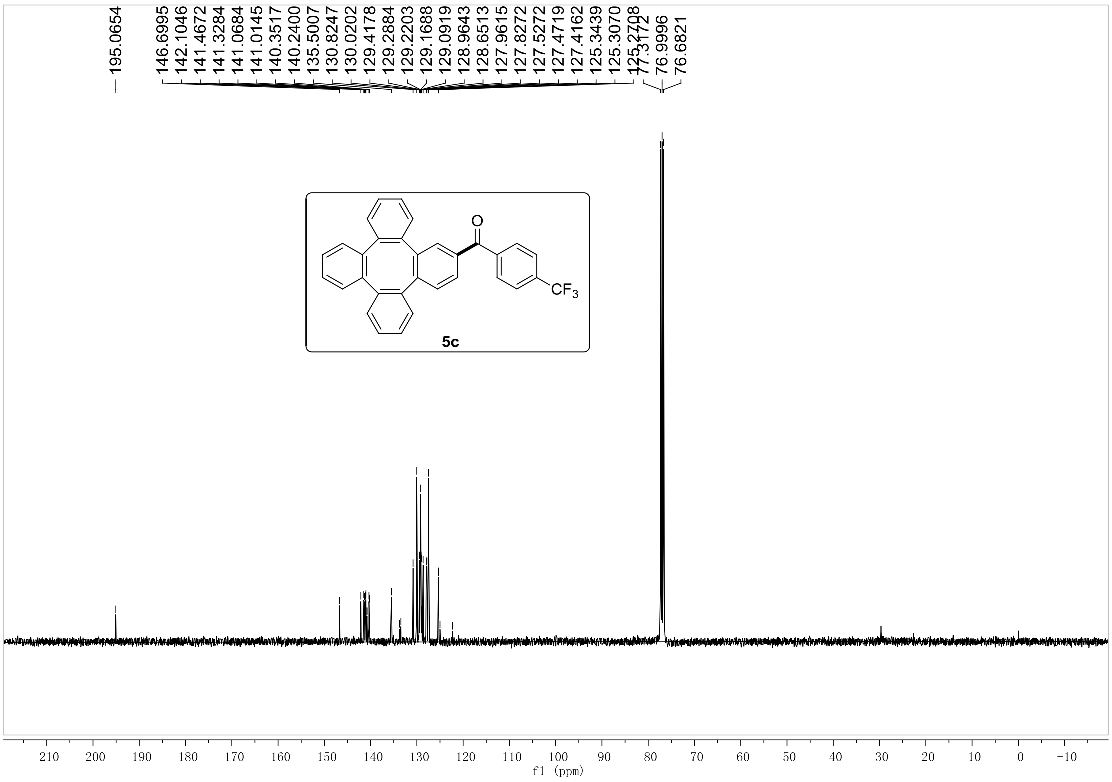

^{1}H NMR-5d

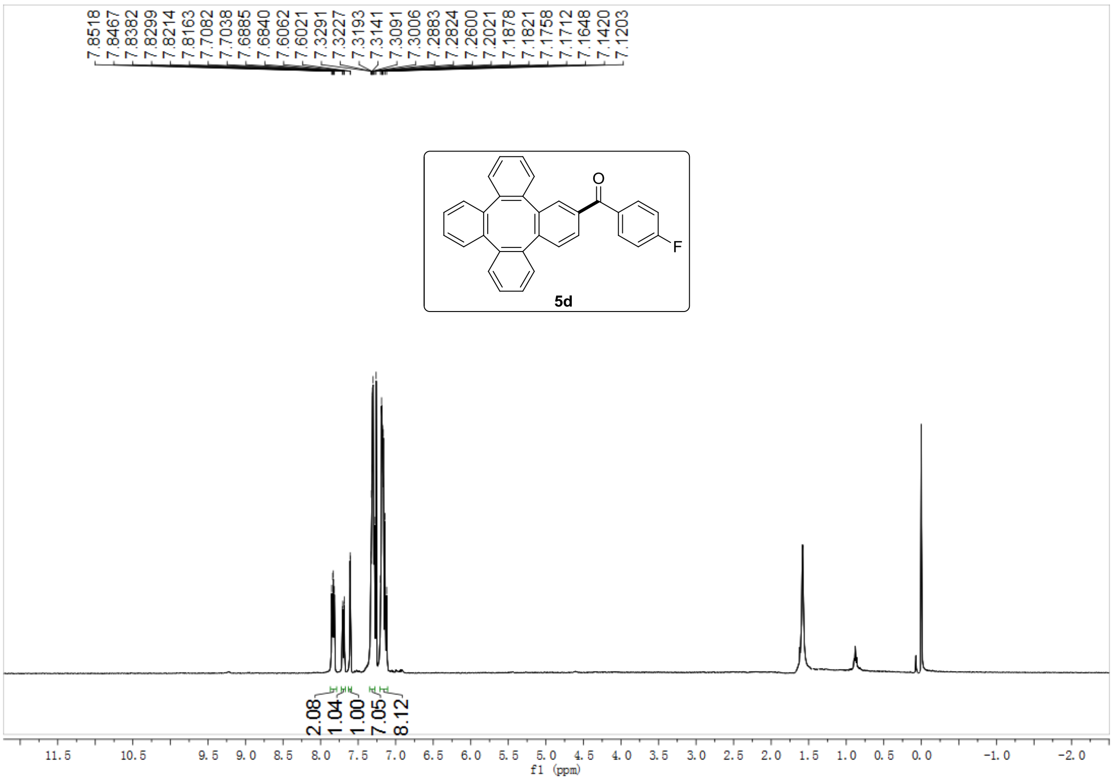

^{13}C NMR-5d

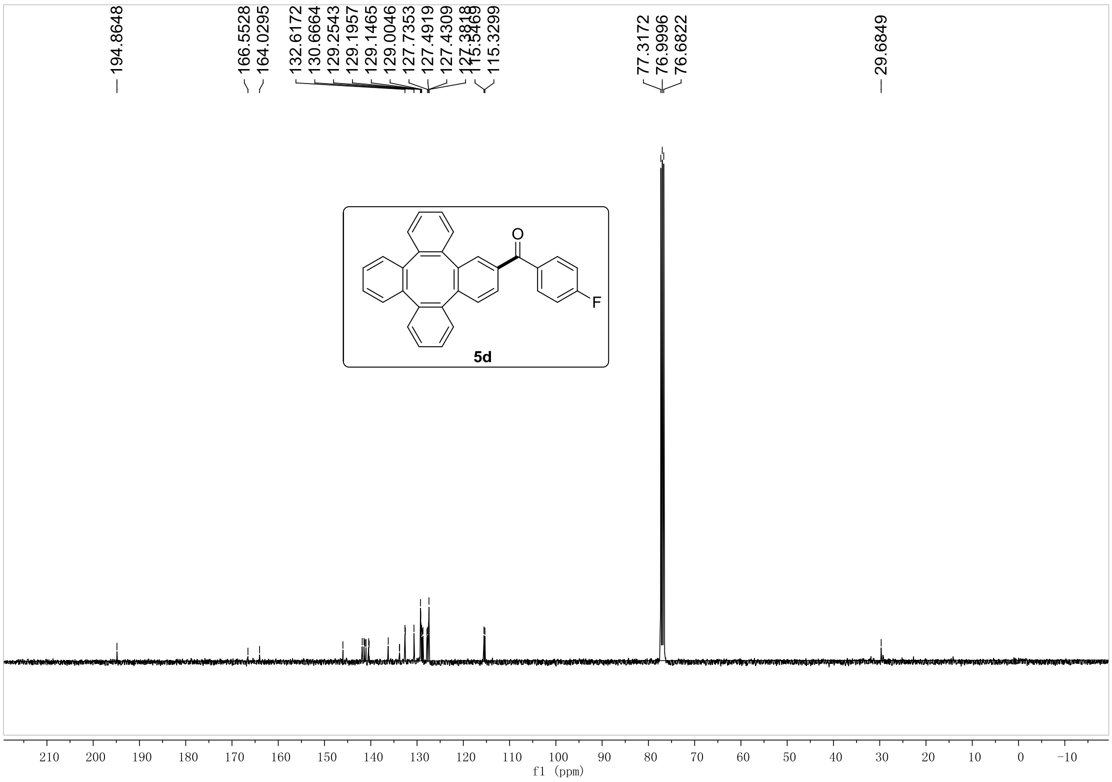

1H NMR-5e

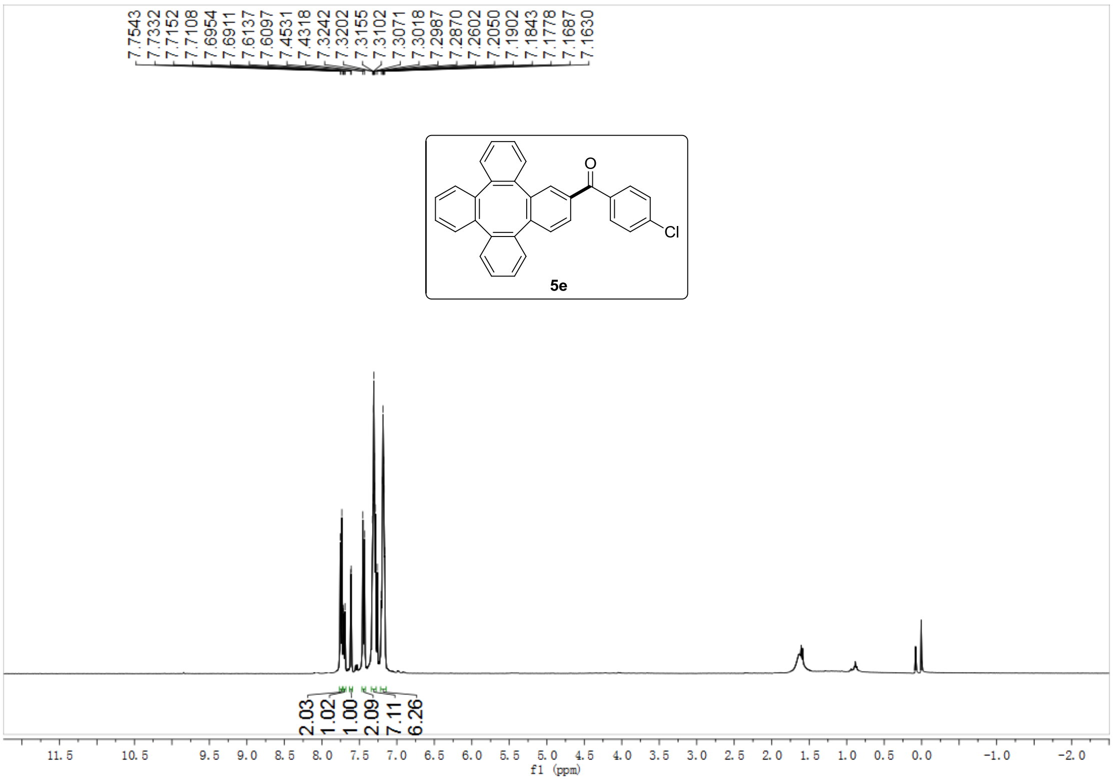

13C NMR-5e

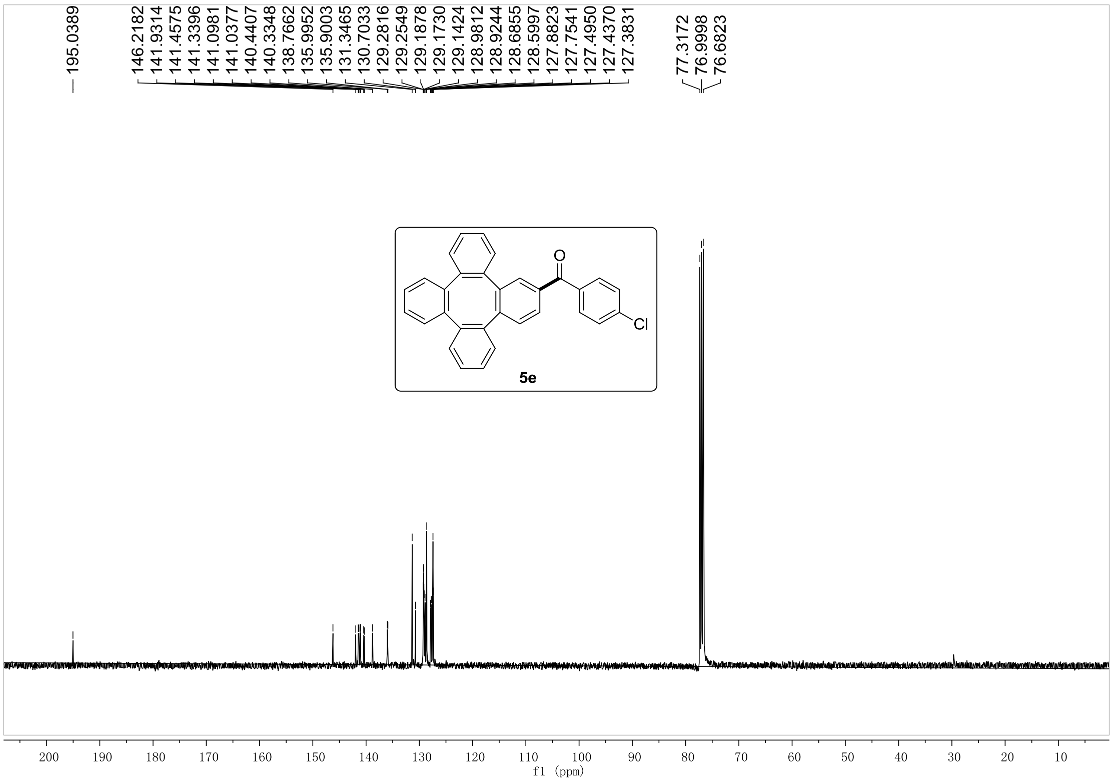

^{1}H NMR-5f

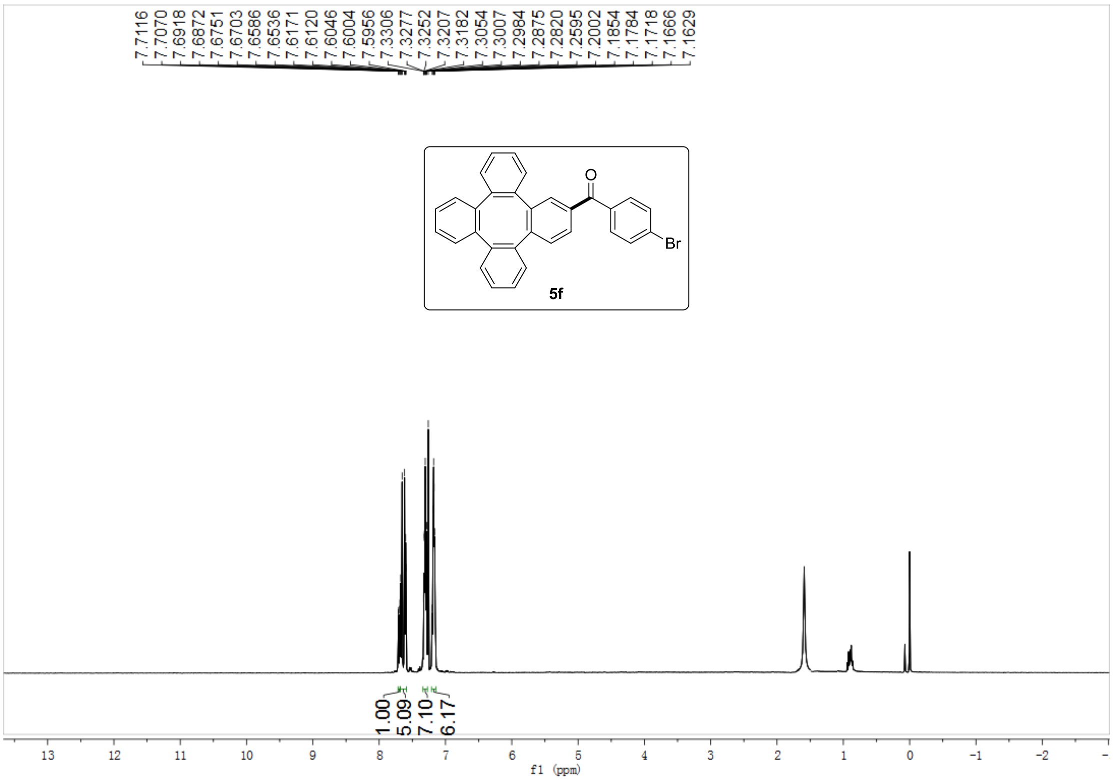

^{13}C NMR-5f

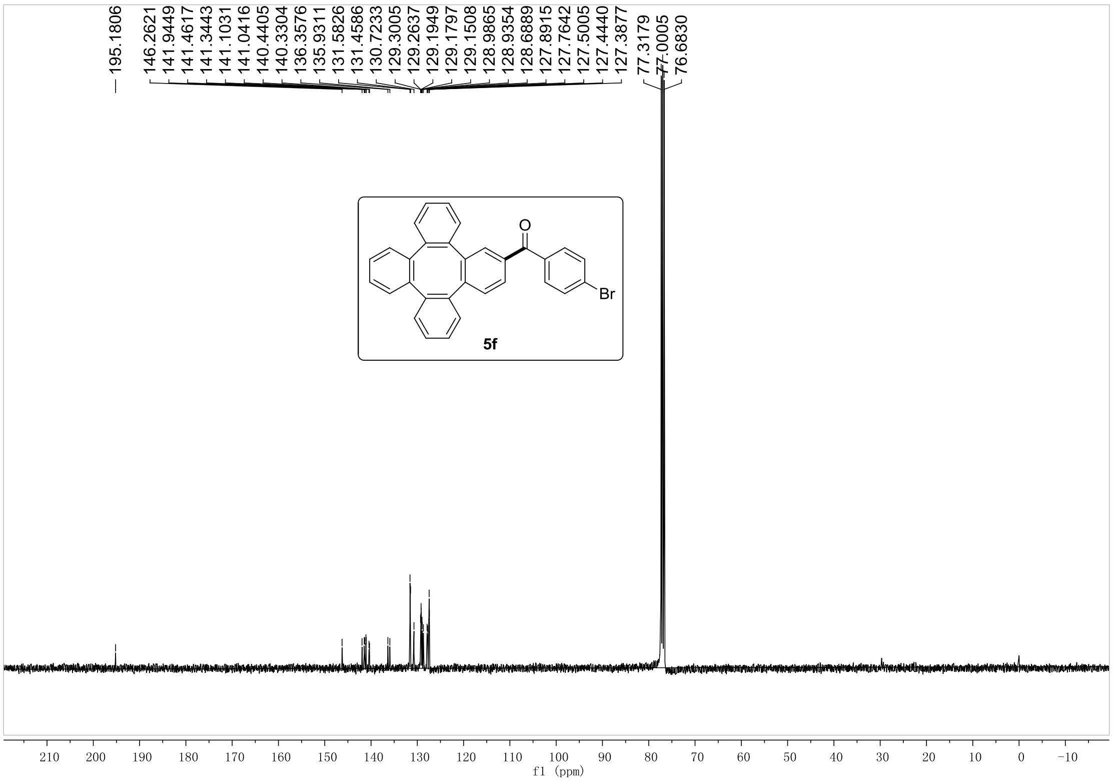

^{1}H NMR-5g

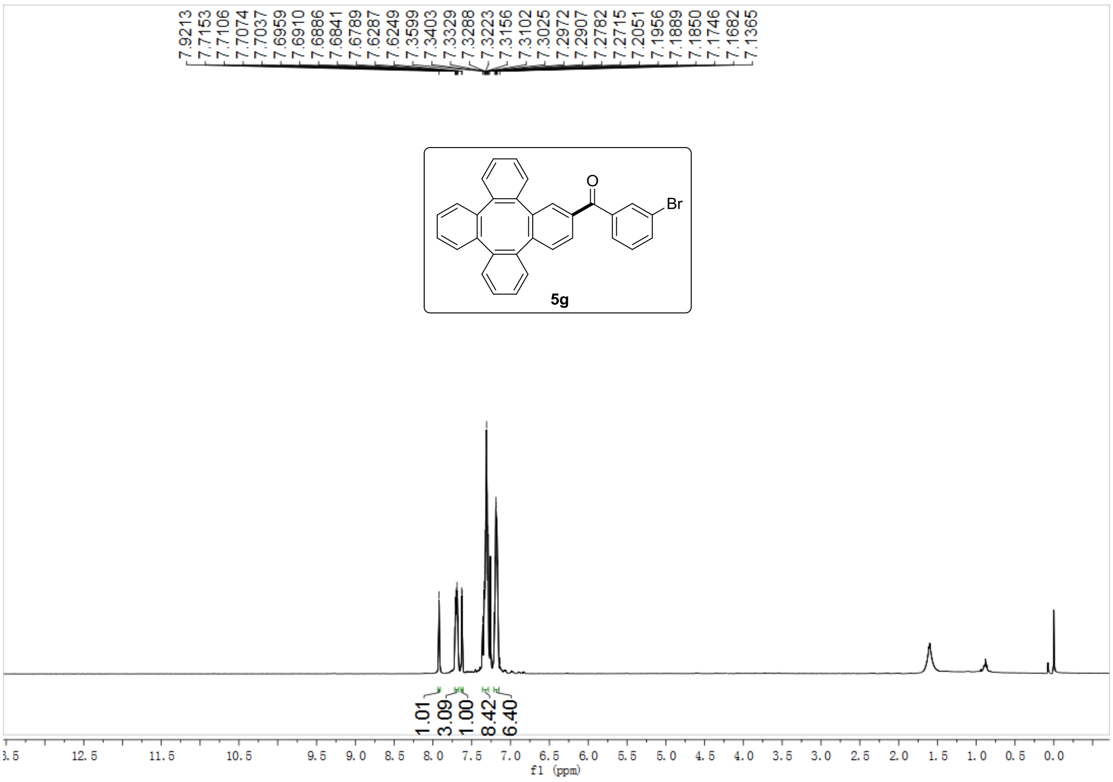

^{13}C NMR-5g

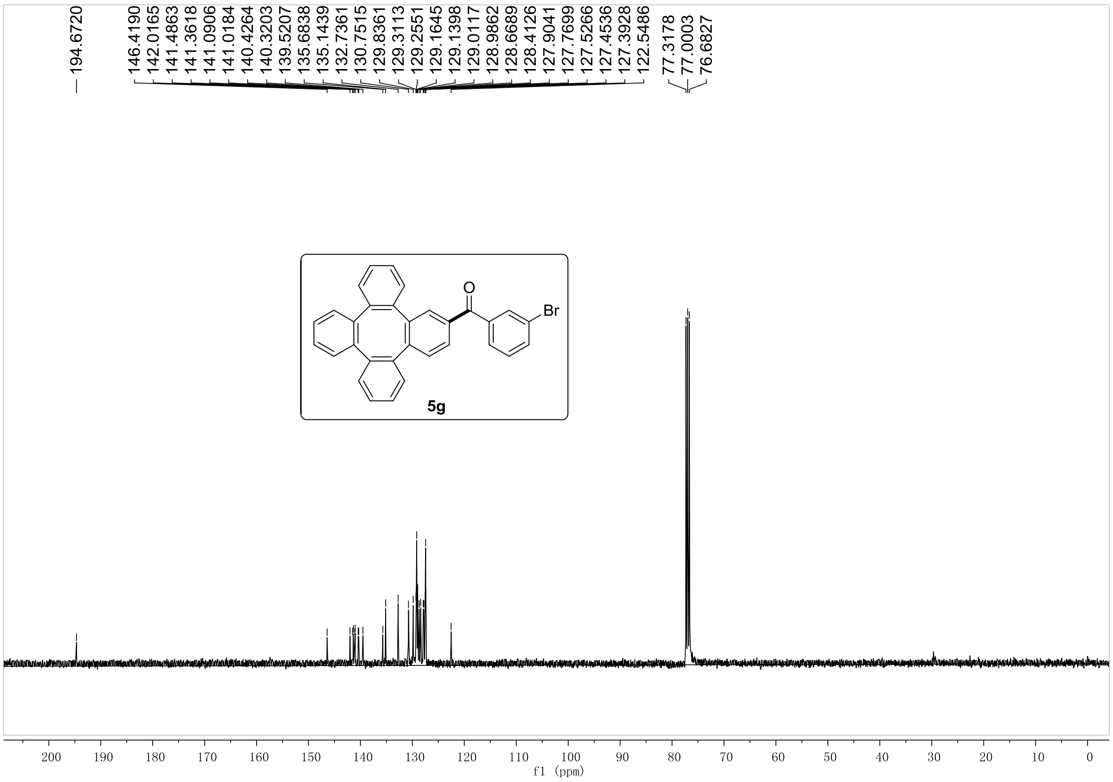

1H NMR-5h

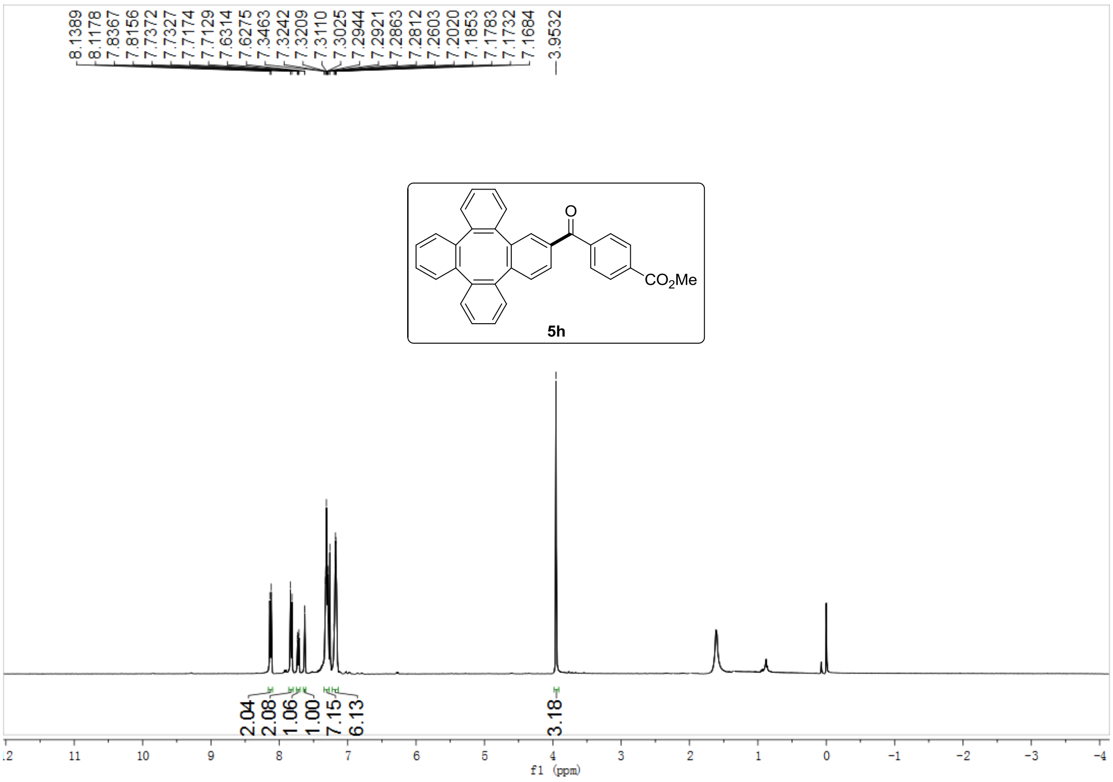

13C NMR-5h

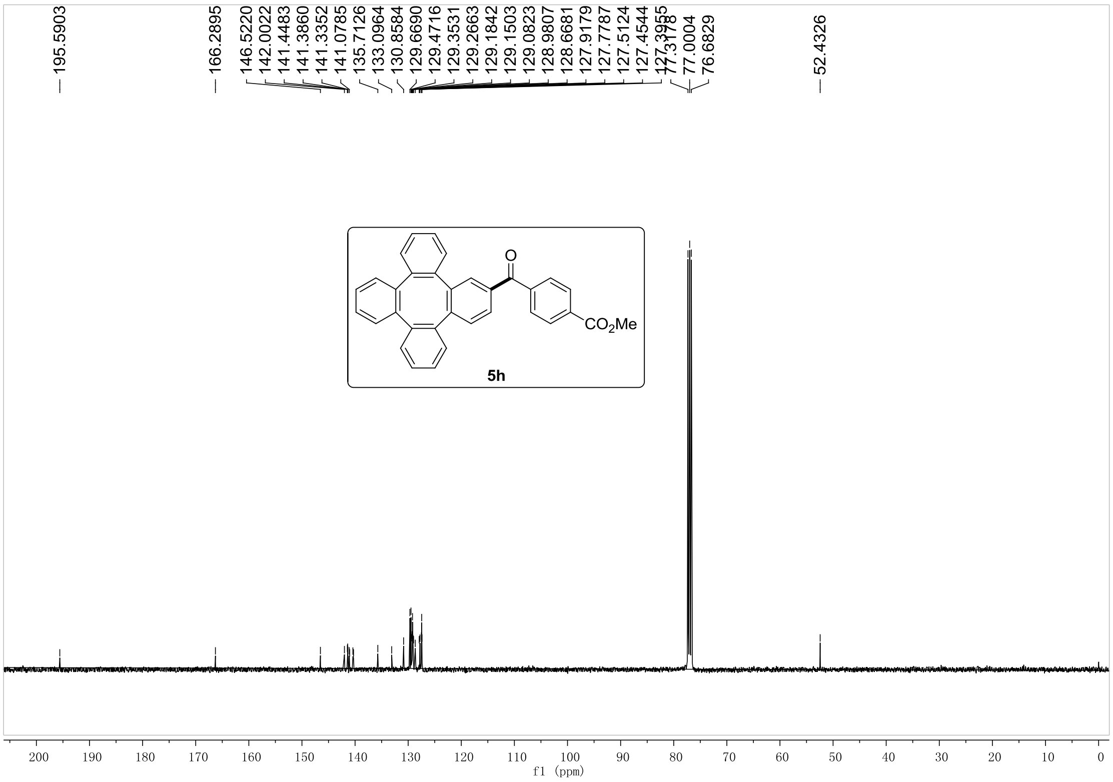

^{1}H NMR-5i

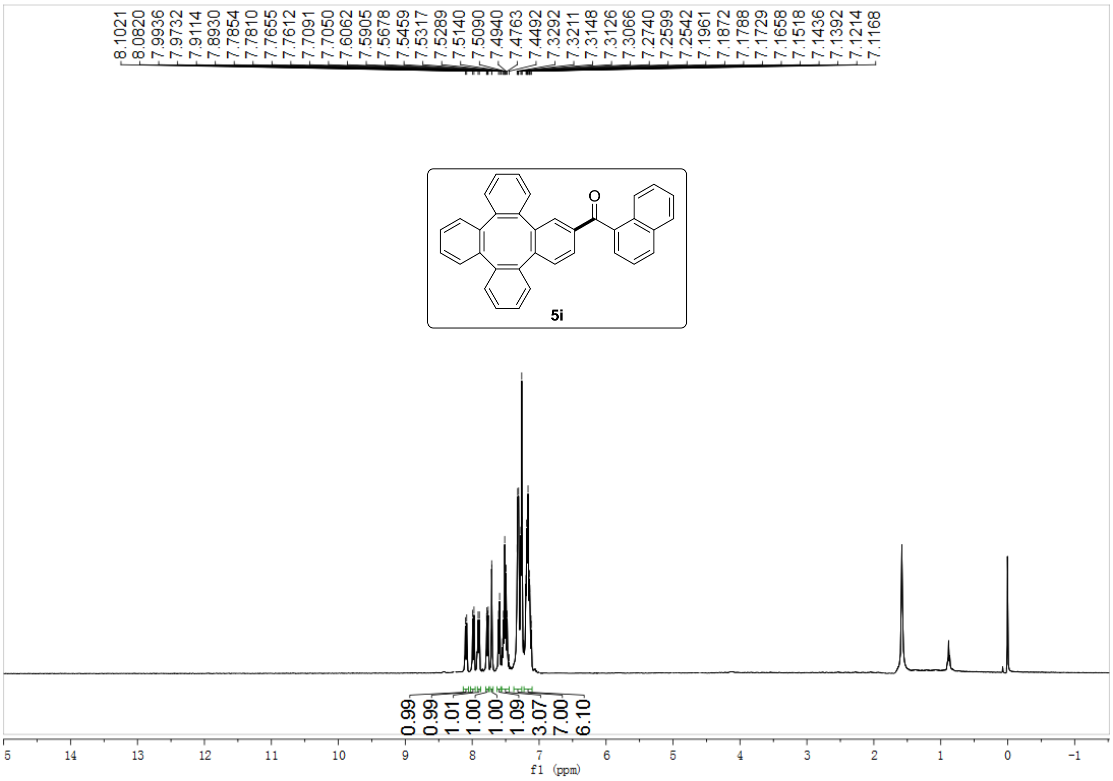

^{13}C NMR-5i

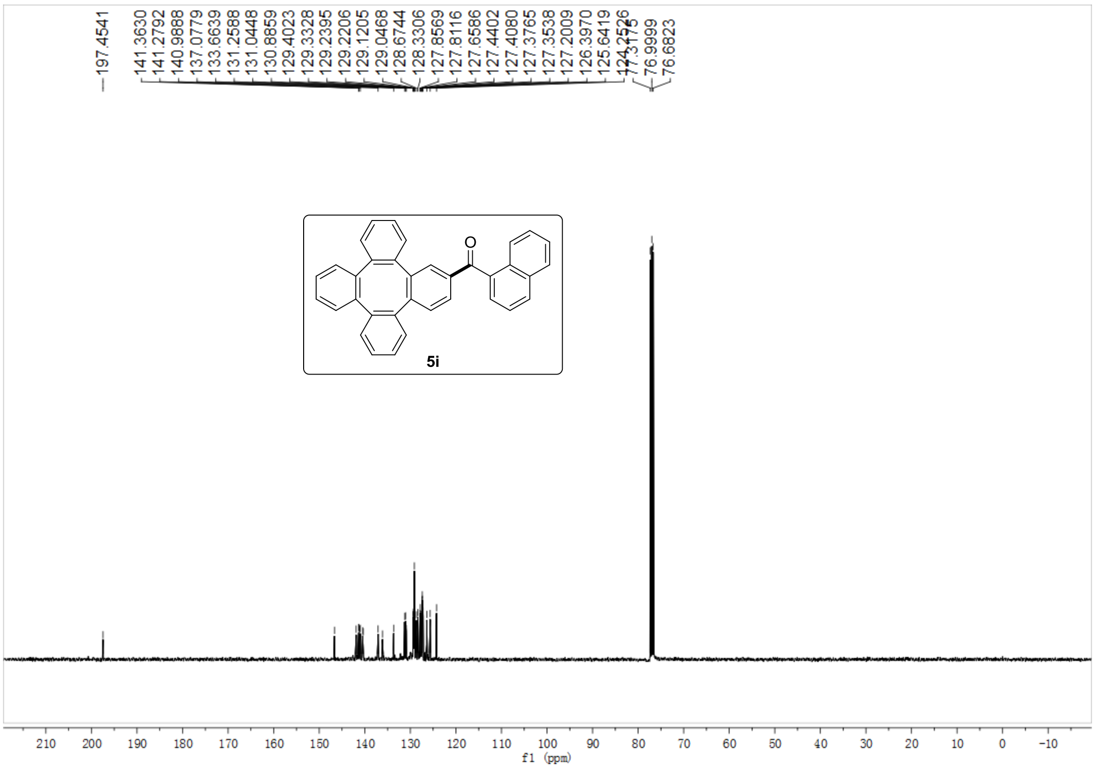

^{1}H NMR-5j

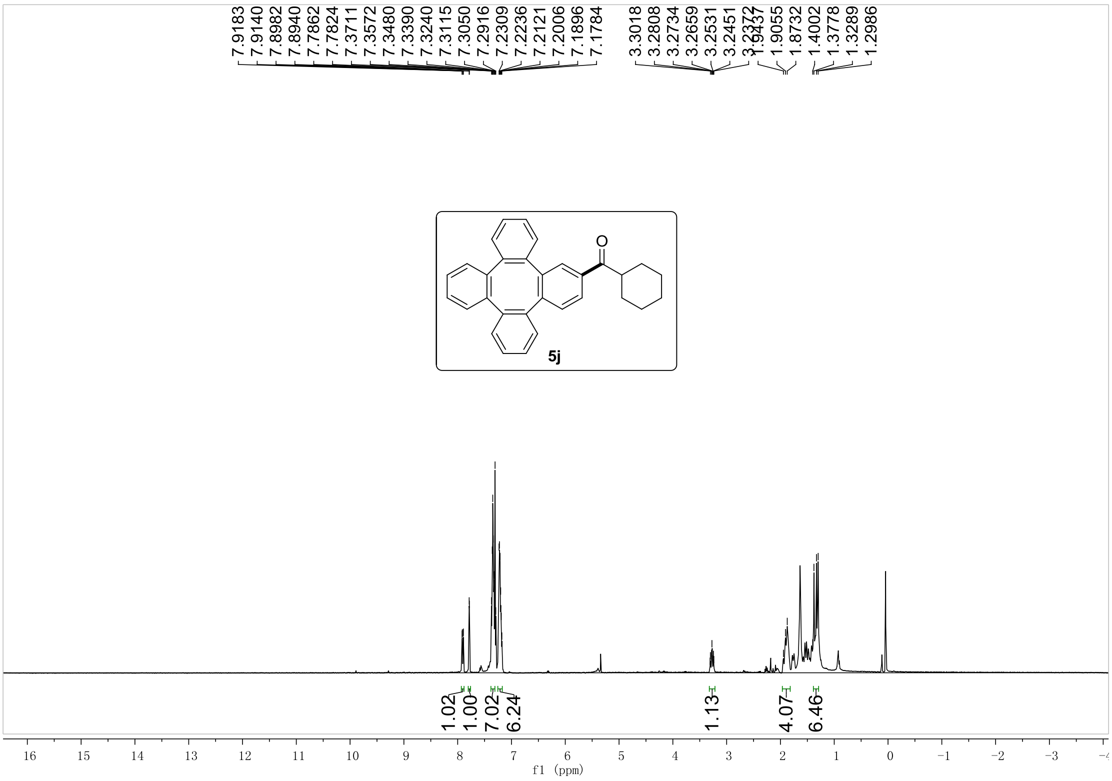

^{13}C NMR-5j

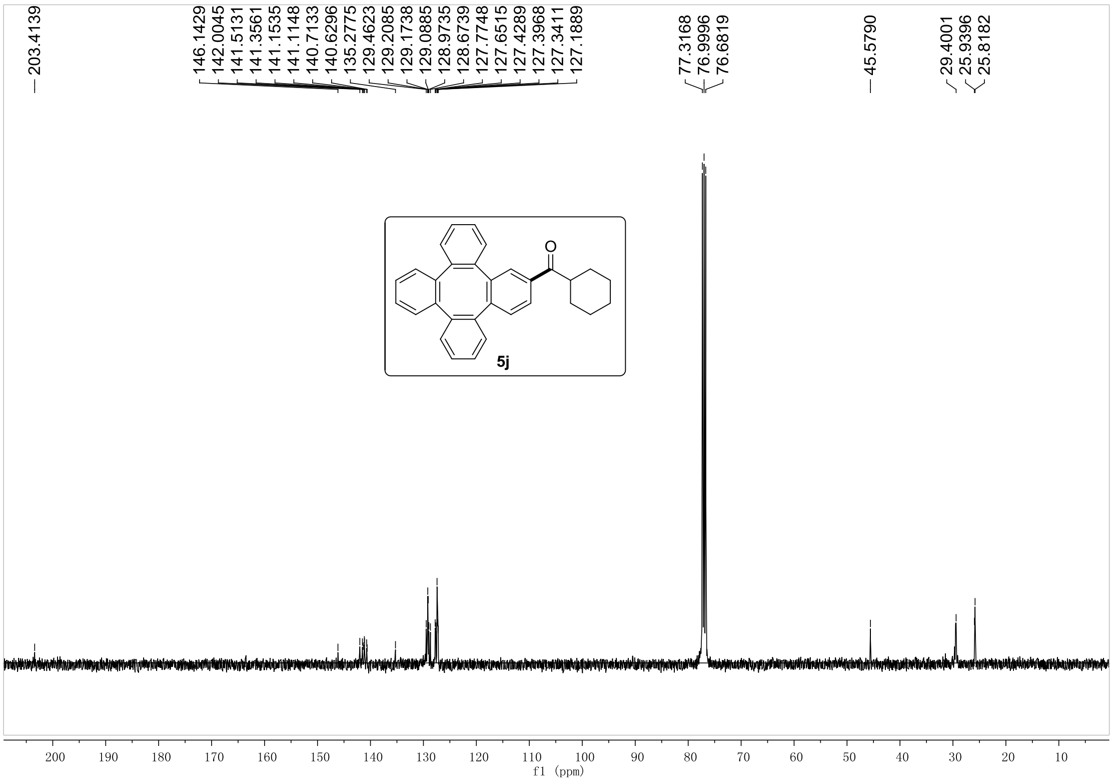

^{1}H NMR-5k

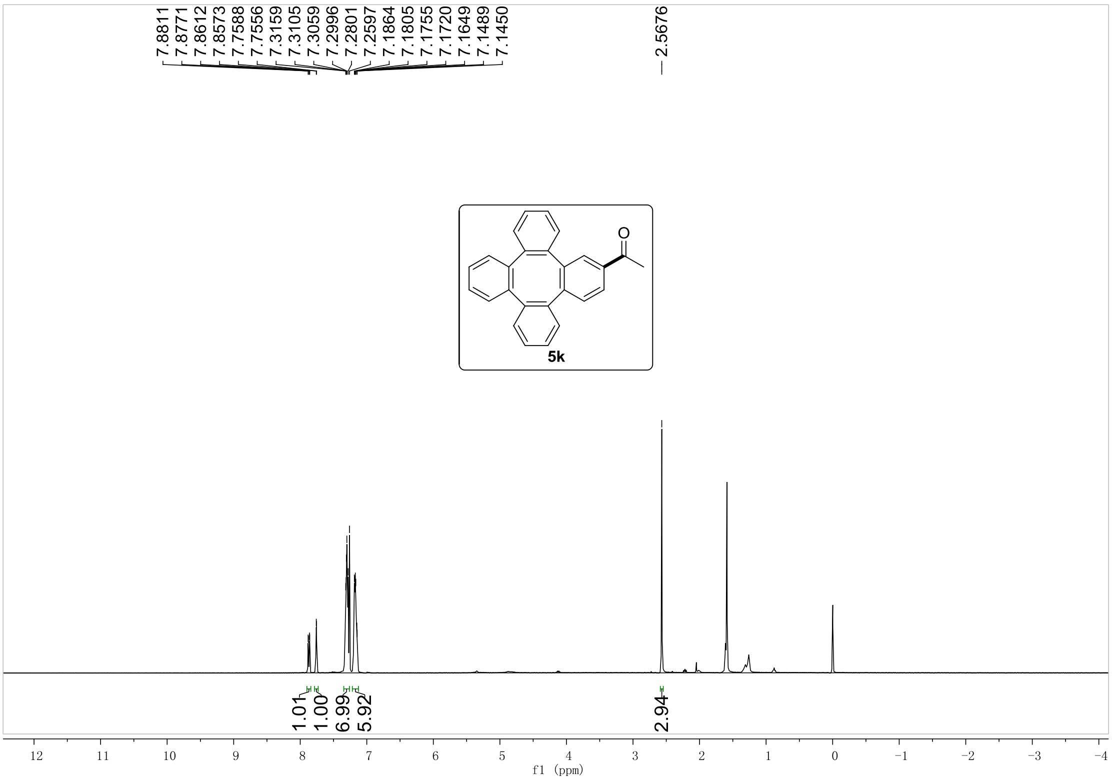

^{13}C NMR-5k

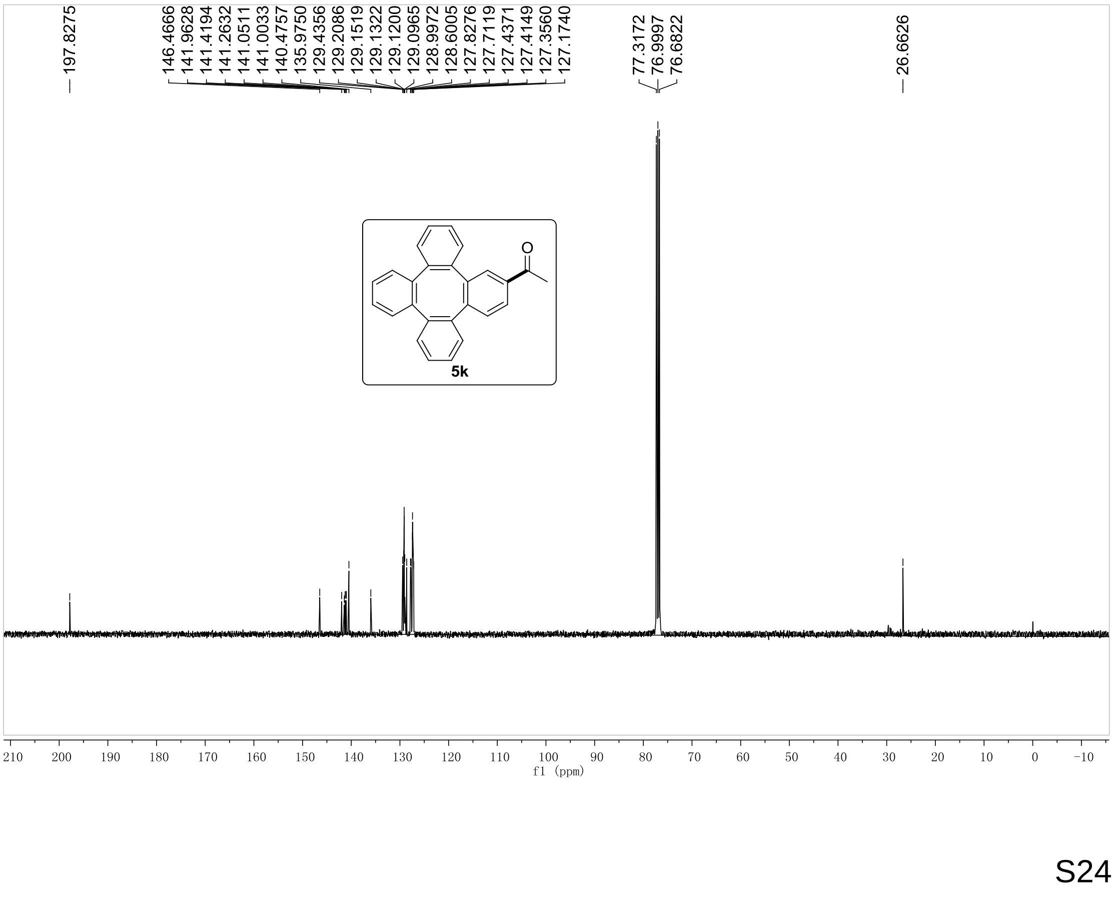

Supplement: File 1 — Experimental section and characterization of the synthesized compounds. [file Beilstein_J_Org_Chem-12-1302-s001.pdf]
